# Pifithrin-μ sensitizes mTOR-activated liver cancer to sorafenib treatment

**DOI:** 10.1038/s41419-025-07332-6

**Published:** 2025-01-26

**Authors:** Jiarui Lv, Yanan Wang, Jiacheng Lv, Cuiting Zheng, Xinyu Zhang, Linyan Wan, Jiayang Zhang, Fangming Liu, Hongbing Zhang

**Affiliations:** 1 Department of Organ Transplantation and Hepatobiliary Surgery, Key Laboratory of Organ Transplantation of Liaoning Province, The First Hospital of China Medical University, Shenyang, China; 2 Department of Physiology, State Key Laboratory of Common Mechanism Research for Major Diseases, Haihe Laboratory of Cell Ecosystem, Institute of Basic Medical Sciences and School of Basic Medicine, Chinese Academy of Medical Sciences and Peking Union Medical College, Beijing, China; 3 Department of Plastic Surgery, The First Hospital of China Medical University, Shenyang, China; 4 Department of Radiology, State Key Laboratory of Complex, Severe and Rare Diseases, Chinese Academy of Medical Sciences, Peking Union Medical College and Peking Union Medical College Hospital, Beijing, China; 5 Department of Gastroenterology, Yichang Central People’s Hospital, The First College of Clinical Medical Science, China Three Gorges University, Yichang, China; 6 Department of Breast Oncology, Key Laboratory of Carcinogenesis and Translational Research, Peking University Cancer Hospital and Institute, Beijing, China

**Keywords:** Cancer therapy, Cell death

## Abstract

TSC2, a suppressor of mTOR, is inactivated in up to 20% of HBV-associated liver cancer. This subtype of liver cancer is associated with aggressive behavior and early recurrence after hepatectomy. Being the first targeted regimen for advanced liver cancer, sorafenib has limited efficacy in HBV-positive patients. In this study, we observed that mTOR-activated cells, due to the loss of either TSC2 or PTEN, were insensitive to the treatment of sorafenib. Mechanistically, HSP70 enhanced the interaction between active mTOR-potentiated CREB1 and CREBBP to boost the transcription of the antioxidant response regulator SESN3. In return, elevated SESN3 enhanced cellular antioxidant capacity and rendered cells resistant to sorafenib. Pifithrin-μ, an HSP70 inhibitor, synergized with sorafenib in the induction of ferroptosis in mTOR-activated liver cancer cells and suppression of TSC2-deficient hepatocarcinogenesis. Our findings highlight the pivotal role of the mTOR-CREB1-SESN3 axis in sorafenib resistance of liver cancer and pave the way for combining pifithrin-μ and sorafenib for the treatment of mTOR-activated liver cancer.

## Introduction

Primary liver cancer is the sixth most common cancer and the third most common cause of cancer-related deaths globally [1, 2]. The number of new cases of hepatic cancer is projected to increase by >55% between 2020 and 2040 [3, 4]. As persistent infection with hepatitis B virus (HBV) is a major risk factor, nearly half of the liver cancer cases diagnosed globally occur in China [3, 5]. Hepatic cancer is the fourth most common cancer and the second leading cause of cancer deaths in China [6]. Hepatocellular carcinoma (HCC) accounts for up to 90% of liver cancer [7]. The majority of HCC cases are diagnosed at advanced stages, resulting in limited treatment efficacy and unfavorable prognoses [8]. Therefore, there is a compelling need to establish better treatment strategies for advanced hepatic cancer.

Sorafenib, a multi-target tyrosine kinase inhibitor, is the first targeted drug for the treatment of advanced HCC [9]. Although sorafenib has been widely used in clinics for stabilizing HCC progression, different etiological factors may contribute to various response rates of cells to sorafenib [10, 11]. While 40% to 60% of HCC patients may benefit from sorafenib initially, this population develops drug resistance within 6 months of treatment [12, 13]. As drug resistance contributes to the limited survival benefits of sorafenib, strategies to overcome both primary and acquired resistance are urgently needed [14]. The documented mechanisms of sorafenib resistance include activation of the hypoxia-induced pathway, epithelial-mesenchymal transition, reduced levels of reactive oxygen species (ROS) and others [15–17]. Under normal conditions, cells maintain a balance between ROS generation and scavenging through oxidation and antioxidation processes. Numerous studies have shown that sorafenib induces ferroptosis by promoting the generation of ROS and iron accumulation [18, 19]. Therefore, strategies aimed at reducing the antioxidant capacity of tumor cells hold the potential for reversing sorafenib resistance.

Mechanistic target of rapamycin (mTOR), a serine/threonine kinase, regulates various cellular processes, including cell metabolism, growth, proliferation, and survival. Loss of tumor suppressor phosphatase and tensin homolog (PTEN) or tuberous sclerosis complex 2 (TSC2) activates mTOR signaling pathway and is associated with various cancers [20–25]. Multiple mTOR signaling pathway components were recurrently mutated in HBV-associated HCC. Up to 59% of HCC patients exhibit mTOR activation, which is linked to early recurrence and poor prognosis [26–28]. PTEN is mutated or silenced in approximately half of primary hepatoma patients [29]. About 6.3% to 20% of Asian HCC are associated with loss of TSC2 function [30–33]. Array-based pathway profiling revealed mTOR activation in sorafenib-resistant HCC cells [34]. Lack of efficacy for Sorafenib has been reported in HBV-positive patients [10]. Therefore, mTOR-activated cells might be resistant to sorafenib. Although mTOR inhibitors are effective in the treatment of benign tumor with mTOR activation [35, 36], their efficacy is limited in malignant tumor [37, 38], largely due to their cytostatic nature. There is no solid evidence suggesting a true benefit of mTOR inhibitors in liver cancer treatment [39]. Moreover, clinical trials of combining sorafenib and mTOR inhibitor everolimus did not improve overall survival of HCC patients [40, 41].

In this study, we found that mTOR-activated cells were resistant to sorafenib treatment. Antioxidant protein sestrin 3 (SESN3) was a novel effector of mTOR-cAMP responsive element-binding protein 1 (CREB1) signaling cascade. mTOR activation-mediated sorafenib resistance resulted from the accumulation of SESN3 and consequent enhancement of antioxidant capacity. Pifithrin-μ, also known as 2-phenylethyenesulfonamide (PES), is a heat shock protein 70 (HSP70) inhibitor that disrupted the interaction between CREB1 and CREB-binding protein (CREBBP), ultimately suppressed SESN3 to enhance the anti-tumor effects of sorafenib. A combination of pifithrin-μ and sorafenib may thus represent a novel therapeutic approach to overcome sorafenib resistance in mTOR-activated liver cancer.

## Materials and methods

### Reagents

Sorafenib (#HY-10201) and SGC-CBP30 (#HY-15826) were purchased from MedChemExpress (MCE, Monmouth Junction, NJ, USA). Deferoxamine mesylate (DFO, #D9533) was obtained from Sigma-Aldrich (St. Louise, MO, USA). Ferrostatin-1 (Fer-1, #S7243), N-acetylcysteine (NAC, #S1623), necrostatin-1 (Nec-1, #S8037), chloroquine (CQ, #S6999) and pifithrin-μ (PES, #S2930) were purchased from Selleck Chemicals (Houston, TX, USA).

### Cell lines and culture

Wild-type (WT) mouse embryonic fibroblasts (MEFs), *Tsc2*^*−/−*^ MEFs, *Pten*^*−/−*^ MEFs, 293FT, HCCLM3, HepG2, MHCC97H, SNU886, and SNU398 cell lines have been previously described [42–44]. The Human Li7 cell line was generously provided by Huang Lin (Dalian Medical University, Dalian, China). These cell lines were cultured in DMEM (#11995-065, Gibco, Grand Island, NY, USA) or RPMI 1640 (#72400047, Gibco) supplemented with 10% FBS (#10100147 C, Gibco) and 1% penicillin-streptomycin (#15140163, Life Technologies, Carlsbad, CA, USA) under 5% CO_2_ at 37 °C.

### Cell viability

MEFs and liver cancer cells were seeded at a density of 3 × 10^3^ cells per well in 96-well plates. After treatment with various compounds for a specific time, 10 μL of CCK8 solution (#40203ES60, Yeasen, Shanghai, China) were added to each well and incubated for 2 h. The absorbance at 450 nm was detected using a Thermo Multiskan MK3 Microplate reader (Thermo Fisher Scientific, Waltham, MA, USA).

### Intracellular ROS and lipid ROS measurement

The levels of intracellular ROS and lipid peroxidation were quantified using DCFH-DA (#S0033M, Beyotime, Shanghai, China) and C11-BODIPY (#D3861, Thermo Fisher Scientific), respectively, following the manufacturer’s instructions. In brief, cells were incubated with probes for 20 min at 37 °C in a light-shielded environment. Subsequently, cells were collected and washed with PBS, and the fluorescence intensity was measured using a CytoFlex flow cytometer (Beckman-Coulter, Fullerton, CA, USA). Data analysis was performed using FlowJo version 10 software.

### Plasmid constructions and lentiviral infection

pcDNA3-Flag-mTOR was generously provided by Jie Chen (plasmid #26603, Addgene, Cambridge, MA, USA) [45]. Open reading frame human CREB1 and SESN3 cDNAs were subcloned into pcDNA3 plasmids, respectively. Short hairpin RNAs (shRNAs) targeting human mTOR, CREB1, and SESN3 were synthesized and subsequently cloned into pLKO.1 lentiviral vector, respectively. Virus particles produced in 293FT cells were harvested, filtered, and then infected into cells supplemented with polybrene. Cells were transfected with plasmids, shRNAs and HSP70 siRNAs using Lipofectamine 2000 (#11668019, Invitrogen, Carlsbad, CA, USA). The corresponding sequences for shRNAs/siRNAs are provided below:

sh-*mTOR*-1:5’-CCGCTAGTAGGGAGGTTTATT-3′

sh-*mTOR*-2:5’-CCTGGCAACAATAGGAGAATT-3′

sh-*CREB1*-1:5’-GCTCGATAAATCTAACAGTTA-3′

sh-*CREB1*-2:5’-GCAAACATTAACCATGACCAA-3′

sh-*SESN3*-1:5’-GCTGAACTTCTTTATGCTCTT-3′

sh-*SESN3*-2:5’-CAGTTCTCTAGTGTCAAAGTT-3′

si-*HSP70*: 5’-GCCTTTCCAAGATTGCTGT-3′

### Immunoblotting

Cellular proteins were extracted using SDS sample buffer supplemented with a Protease and Phosphatase Inhibitor Cocktail (New Cell & Molecular Biotech, Suzhou, China). The samples were denatured, separated by SDS-polyacrylamide gel electrophoresis (SDS-PAGE), and then transferred onto nitrocellulose membranes. After blocking with milk, the membranes were incubated with primary antibodies as follows: β-actin (#sc-47778, Santa Cruz Biotechnologies, Santa Cruz, CA, USA), TSC2 (#4308, Cell Signaling Technologies [CST], Danvers, MA, USA), mTOR (#2983, CST), 4EBP1 (#9644, CST), P-4EBP1 (#2855, CST), P70S6K (#2708, CST), P-P70S6K (#9205, CST), PTEN (#9559, CST), P-AKT (#4060, CST), AKT (#4691, CST), PDK1(#3062, CST), CREB1 (#9197, CST), CREBBP (#7389, CST), HSP70 (#M20033, Abmart, Shanghai, China), SESN3 (#11431-2-AP, Proteintech, Rosemont, IL, USA), TFRC (#ab214039, Abcam, Cambridge, UK) and Ferritin (#T55648, Abmart). Subsequently, the membranes were probed with IRDye secondary antibodies (#32210/#68071, LI-COR Biosciences, Lincoln, NE, USA) and imaged using LI-COR Odyssey CLX.

### Coimmunoprecipitation (Co-IP) assay

Protein extracts were incubated with antibodies-coupled magnetic beads (#HY-K0202, MCE) to pull down CREB1 (#9197, CST), CREBBP (#7389, CST), or HSP70 (#M20033, Abmart) at 4 °C overnight. IgG (#32935, CST) served as a negative control for antibodies. The beads were washed with Co-IP buffer three times and then boiled in SDS loading buffer at 98°C for 10 minutes. Subsequently, the proteins were subjected to SDS-PAGE analysis.

### Luciferase reporter assay

For luciferase reporter assays, cells (5 × 10^4^ cells per well) were seeded in a 24-well culture plate and incubated overnight. Renilla construct and reporter constructs cloned in pGL3-basic vector (#E1751, Promega Corporation, Madison, WI, USA) were co-transfected with either CREB1 overexpression plasmid or corresponding negative controls, using Lipofectamine 2000. After 48 h, luciferase activity was quantified using Dual Luciferase Reporter Gene Assay Kit (#11402ES, Yeasen) and normalized to Renilla luciferase activity according to the manufacturer’s instructions. The primers for human SESN3 gene were as follows: Response element 1 (RE1), 5’-ATCCTGGTACGCTGGAGACC-3’ and 5’-CTTGCATCGCCTACTGGCAA-3’; Response element 1 (RE2), 5’-GGAGACCTGGCTCCCCTAC-3’ and 5’-GCCCTGCTCAGAAAGGAAGG-3’.

### Chromatin-immunoprecipitation (ChIP) assay

The ChIP assay was performed using an Enzymatic Chromatin IP Kit (#9005, CST). Chromatin extracts containing DNA fragments were isolated through immunoprecipitation using CREB1 antibody (#9197, CST), subjected to qPCR using the previously described primers, and DNA enrichment was calculated relative to the total input chromatin: 2% × 2^(C[T] [2%Input] Sample − C[T] IP Sample)^.

### Measurement of total antioxidant capacity, NADPH/NADP+ ratio and GSH level

The total antioxidant activity was determined using a rapid 3-ethylbenzthiazoline-6-sulfonic acid (ABTS) assay (#S0121, Beyotime) following the manufacturer’s protocol. ABTS is oxidized to green ABTS•^+^ in the presence of suitable oxidants. The antioxidant molecules in cell lysates inhibited ABTS radical scavenging activity which was measured by recording the absorbance at 414 nm. The total antioxidant capacity of the samples was determined from a standard curve and reported as Trolox concentration. The NADP + /NADPH Assay Kit with WST-8 (#S0179, Beyotime) and the Total Glutathione Assay Kit (#E2015, Pplygen, Beijing, China) were used to assess NADPH/NADP+ ratio and total GSH level, respectively, in accordance with the manufacturers’ instructions. Cell lysates were evaluated by measuring the absorbance at 450 nm or 412 nm. All results were normalized based on protein content.

### RNA extractions and real-time quantitative PCR

Total RNA was extracted from cells using TRIZOL reagent (Invitrogen). The RNA was reverse transcribed into cDNA using the Hifair® II 1st Strand cDNA Synthesis Kit (#11121ES60, Yeasen). qRT-PCR analysis was performed to quantify gene expression using SYBR qPCR Mix (#abs60086, Absin Biosciences, Shanghai, China). The sequences of the PCR primers used are as follows:

Human *β-actin* Forward 5’-AGAGGGAAATCGTGCGTGAC-3’

Human *β-actin* Reverse 5’-CAATAGTGATGACCTGGCCGT-3’;

Human *CREB1* Forward 5’-GACCACTGATGGACAGCAGATC-3’

Human *CREB1* Reverse 5’-GAGGATGCCATAACAACTCCAGG-3’;

Human *SESN3* Forward 5’-GACAGTGACCTGCTATCCTGAG-3’

Human *SESN3* Reverse 5’-CCGAGTTATGGCACGAAGAGCA-3’;

Human *BCL2* Forward 5’-ATCGCCCTGTGGATGACTGAGT-3’

Human *BCL2* Reverse 5’-GCCAGGAGAAATCAAACAGAGGC-3’;

Human *BDNF* Forward 5’-CATCCGAGGACAAGGTGGCTTG-3’

Human *BDNF* Reverse 5’-GCCGAACTTTCTGGTCCTCATC-3’;

Human *ATF3* Forward 5’-CGCTGGAATCAGTCACTGTCAG-3’

Human *ATF3* Reverse 5’-CTTGTTTCGGCACTTTGCAGCTG-3’;

Human *PDK1* Forward 5’-CATGTCACGCTGGGTAATGAGG-3’

Human *PDK1* Reverse 5’-CTCAACACGAGGTCTTGGTGCA-3’;

Mouse *β-actin* Forward 5’-CATTGCTGACAGGATGCAGAAGG-3’

Mouse *β-actin* Reverse 5’-TGCTGGAAGGTGGACAGTGAGG-3’;

Mouse *Sesn3* Forward 5’-GCGCATGTATGACAGCTACTGG-3’

Mouse *Sesn3* Reverse 5’-TCAGATGCCGAGTTATGGCTCG-3’;

Mouse *Pdk1* Forward 5’-CCACTGAGGAAGATCGACAGAC-3’

Mouse *Pdk1* Reverse 5’-AGAGGCGTGATATGGGCAATCC-3’;

Mouse *Gpx7* Forward 5’-CGACTTCAAGGCGGTCAACATC-3’

Mouse *Gpx7* Reverse 5’-AAGGCTCGGTAGTTCTGGTCTG-3’;

Mouse *Ak4* Forward 5’-GAAGCAGTTGCTGCCAGGCTAA-3’

Mouse *Ak4* Reverse 5’-GCCAGATTCTGTTAGTCTCCGTC-3’

### Fluorescence Imaging

For co-localization, cells were fixed with 4% paraformaldehyde (PFA, #G1101, Servicebio, Wuhan, China) for 20 min, washed three times with PBS, and then treated with blocking buffer (5% BSA, 0.2% Triton X-100 in PBS) for 1 h. After incubation with CREB1 (#9197, CST), CREB1 (#9104, CST), HSP70 (#MA9192S, Abmart) or CREBBP (#TP51023, Abmart) at 4 °C overnight, coverslips were washed with PBS and stained with Alexa-488/Cy3 fluorogenic secondary antibodies (#GB22403/#GB21401, Servicebio) for 2 h at room temperature. For intracellular iron content analysis, cells were fixed in 4% PFA after pretreatment with Phen Green^TM^ SK (PGSK) fluorescence probe (#P14313, Thermo Fisher Scientific) and washed with PBS three times. Images were captured using a stimulated emission depletion (STED) microscope (Leica SP8 STED, Wetzlar, Germany).

### Animal study

All animal experiments were approved by the Animal Research Committee, Institute of Basic Medical Sciences, Chinese Academy of Medical Sciences & Peking Union Medical College. For subcutaneous xenograft study, 2 × 10^6^ HCCLM3 cells transfected with vector or mTOR plasmid, or 3 × 10^6^ SNU886 cells, were subcutaneously injected into the posterior flanks of BALB/c nude mice (female; aged 6 weeks; procured from HFK Bio-Technology, Beijing, China). Once tumor volume reached around 100 mm^3^, mice were randomized into 4 groups (n = 6/group): vehicle, sorafenib (20 mg/kg, i.g.), pifithrin-μ (10 mg/kg, i.p.) and a combination of sorafenib (20 mg/kg, i.g.) and pifithrin-μ (10 mg/kg, i.p.) every other day. Body weights and tumor volumes were measured every 3 days. Tumor volumes were calculated using formula: *V* = (length × width^2^) × 0.5. For spontaneous liver cancer study, *Tsc2* ^flox/flox^ (stock no. 027458) and *Alb-Cre* (stock no. 003574) mice were obtained from Jackson Laboratory (Bar Harbor, Maine, USA). 8-month-old *Tsc2* ^flox/flox^; *Alb*^*Cre*^ mice (a.k.a. *Tsc2*^−/−^) were randomly divided into 4 groups (*n* = 8/group): vehicle, sorafenib (20 mg/kg, i.g.), pifithrin-μ (10 mg/kg, i.p.), and combined sorafenib (20 mg/kg, i.g.) and pifithrin-μ (10 mg/kg, i.p.) every other day for 2 months. Body weights were monitored every 5 days.

### Database analysis

Microarray dataset GSE21755 was obtained from Gene Expression Omnibus database (GEO, http://www.ncbi.nlm.nih.gov/geo) [46]. Differentially expressed gene (DEG) analysis was performed using GEO2R online software. Gene ontology (GO) enrichment analysis was conducted with DAVID functional annotation clustering tool (https://david.ncifcrf.gov/). Genecards database (https://www.genecards.org/) was used to acquire genes involved in protection against oxidative stress with the potential to regulate cellular sorafenib resistance. We obtained SESN3 promoter sequence, which is 2 kb upstream of transcription start site (TSS) from UCSC genome browser (https://genome.ucsc.edu/) using GRCh38/hg38 assembly. JASPAR Transcription Factor Binding Site database (https://jaspar.genereg.net/) was used to predict potential transcription factor of SESN3, generate sequence logo of potential transcription factor and analyze potential response elements on SESN3 promoter sequence. STRING database (https://cn.string-db.org/) was used to conduct protein-protein interaction (PPI) network analysis.

### Statistical analysis

Data were presented as mean ± SD. All data were repeated three times, and statistical analyses were performed using a two-tailed *t* test or one-way analysis of variance in GraphPad Prism version 8 (n.s., not statistically significant. *p < 0.05, **p < 0.01, ***p < 0.001).

## Results

### mTOR activation confers cell resistance to sorafenib

**Fig. 1:**
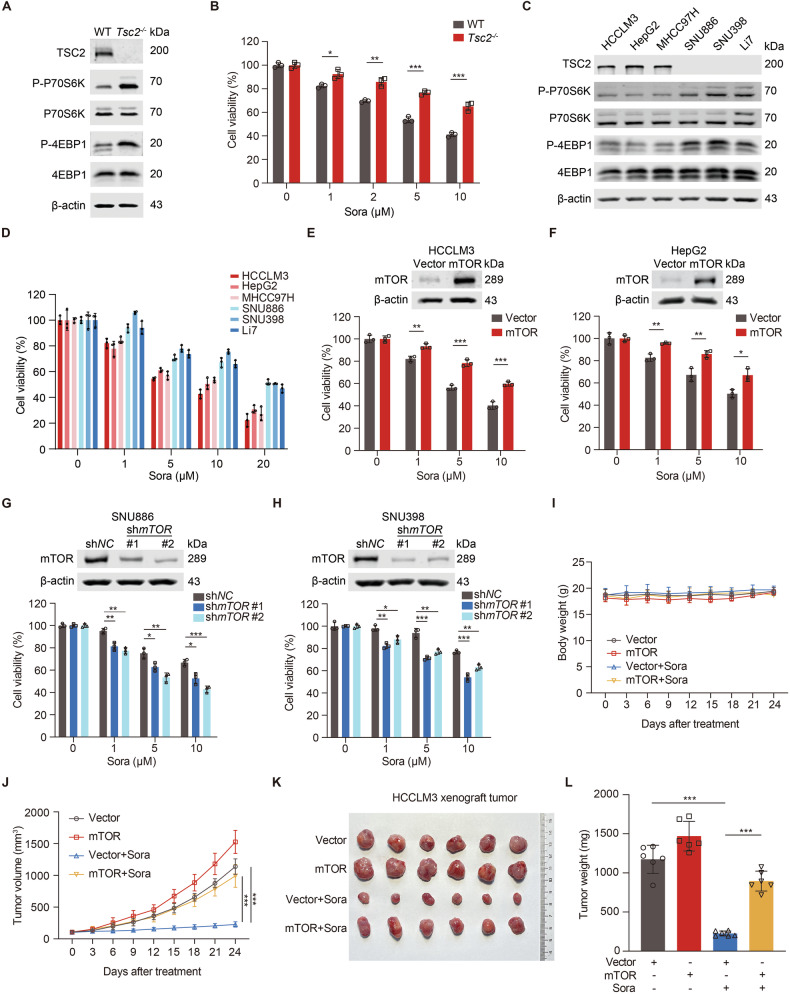
mTOR activation confers cell resistance to sorafenib. **A** Immunoblotting of *Tsc2*^−/−^ MEFs. **B** Viability of *Tsc2*^−/−^ MEFs treated with different concentrations of sorafenib for 24 h, *n* = 3. **C** Immunoblotting of human liver cancer cell lines. **D** Viability of liver cancer cells treated with sorafenib for 24 h, *n* = 3. **E**–**H** CCK8 analysis of HCCLM3 (**E**) or HepG2 (**F**) cells transfected with vector or mTOR plasmid, SNU886 (**G**) or SNU398 (**H**) cells transfected with control or mTOR shRNA, followed by treatment with sorafenib for 24 h, *n* = 3. **I**–**L** Nude mice were xenografted with HCCLM3 cells transfected with vector or mTOR plasmid. Once tumor volume reached ~100 mm^3^, mice were treated with vehicle or sorafenib (20 mg/kg, i.g.) every other day (*n* = 6 per group). Measurements of body weights (**I**) and tumor volumes (**J**) of nude mice every 3 days. Representative images of tumors (**K**) and tumor weights (**L**) were plotted at the end of treatment. Data are displayed as mean ± SD (error bars). *p < 0.05, **p < 0.01, ***p < 0.001. Sora: sorafenib.

Loss of tumor suppressor TSC2 or PTEN causes constitutive mTOR activation. To investigate sorafenib sensitivity of mTOR-activated cells, we first treated WT and *Tsc2*^−/−^ MEFs with sorafenib. *Tsc2*^−/−^ MEFs were less sensitive to sorafenib (Fig. 1A, B). Compared with WT MEFs, *Pten*^−/−^ MEFs also exhibited insensitivity to sorafenib (Fig. S1A, B). We then assessed the impact of mTOR on sorafenib resistance in human liver cancer cells by using 3 TSC2-deficient cell lines (SNU886, SNU398 and Li7) and 3 TSC2 WT cell lines (HCCLM3, HepG2 and MHCC97H) (Fig. 1C). mTOR-activated cells were less sensitive to sorafenib than WT cells (Fig. 1D). Moreover, overexpressing mTOR conferred resistance to HCCLM3 and HepG2 cells (Fig. 1E, F). In contrast, knockdown of mTOR impaired sorafenib resistance of SNU886 and SNU398 cells (Fig. 1G, H). Furthermore, sorafenib blocked tumorigenesis of HCCLM3 cells more effectively than that of HCCLM3/mTOR cells, manifesting as reduced tumor volumes and tumor weights, with minimal effects on body weights of nude mice (Fig. 1I–L). Taken together, mTOR confers cell resistance to sorafenib.

### mTOR attenuates sorafenib-mediated ROS accumulation and oxidative stress in liver cancer cells

**Fig. 2:**
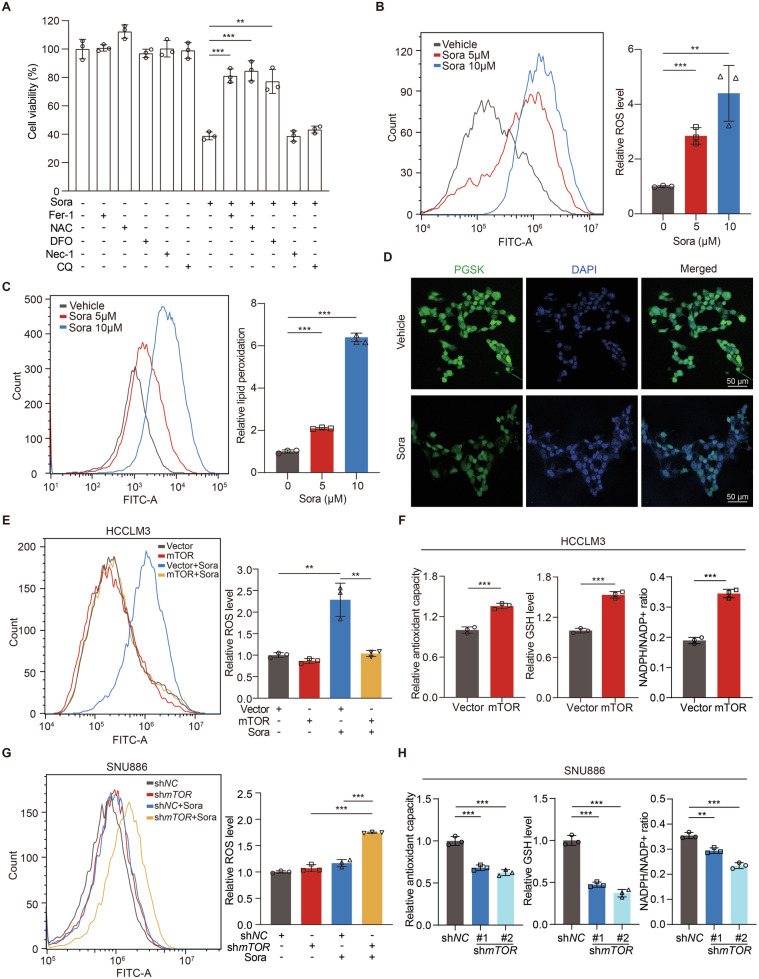
mTOR attenuates sorafenib-mediated ROS accumulation and oxidative stress in liver cancer cells. **A** Viability of HCCLM3 cells treated with sorafenib (10 μM) in the presence or absence of specific inhibitors for 24 h, *n* = 3. Ferrostatin-1 (Fer-1, 10 μM), N-acetylcysteine (NAC, 1 mM), deferoxamine (DFO, 10 μM), necrostatin-1 (Nec-1, 20 μM) and chloroquine (CQ, 10 μM). **B**–**D** HCCLM3 cells were treated with sorafenib (10 μM) for 24 h, *n* = 3. The intracellular ROS (**B**) and lipid peroxidation (**C**) were measured with flow cytometry. Cytosolic Fe^2+^ levels were assayed by PGSK probe, fluorescence intensity was observed under the fluorescence microscope, scale bar=50 μm (**D**). **E**, **F** HCCLM3 cells were transfected with vector or mTOR plasmid and treated with sorafenib (10 μM) for 24 h, *n* = 3. The intracellular ROS were measured with flow cytometry (**E**). Relative total antioxidant capacity, GSH level, and NADPH/NADP+ ratio were measured (**F**). **G**, **H** SNU886 cells were transfected with control or mTOR shRNA and treated with sorafenib (10 μM) for 24 h, *n* = 3. The intracellular ROS were measured with flow cytometry (**G**). Relative total antioxidant capacity, GSH level, and NADPH/NADP+ ratio were measured (**H**). Data are displayed as mean ± SD (error bars). **p < 0.01, ***p < 0.001. Sora: sorafenib.

Sorafenib wields therapeutic effects mainly through the accumulation of lipid ROS and induction of ferroptosis [47]. Consistently, the effect of sorafenib on HCCLM3 cells was partially reversed by Fer-1 (ferroptosis inhibitor), NAC (ROS scavenger) or DFO (iron chelator), but not Nec-1 (necrosis inhibitor) or CQ (autophagy inhibitor) (Fig. 2A). Sorafenib increased intracellular ROS and lipid peroxidation in HCCLM3 cells (Fig. 2B, C). Furthermore, sorafenib raised intracellular Fe^2+^ level, as shown by the quenching of PGSK fluorescence (Fig. 2D, Fig. S2A). As tumor cells often exhibit increased metabolic antioxidant capacity through Warburg effect to promote proliferation [48], we speculated that activation of mTOR facilitates sorafenib resistance through increasing cellular antioxidant capacity of liver cancer cells. Indeed, sorafenib induction of ROS was compromised in HCCLM3/mTOR cells but not HCCLM3 cells (Fig. 2E). Furthermore, total antioxidant capacity, GSH level and NADPH/NADP+ ratio were increased in HCCLM3/mTOR cells as compared to controls (Fig. 2F). Similar findings were also observed in HepG2/mTOR cells (Fig. S2B, C). By contrast, sorafenib increased intracellular ROS level in SNU886/sh*mTOR* cells (Fig. 2G) and SNU398/sh*mTOR* cells (Fig. S2D). Additionally, knockdown of mTOR reduced total antioxidant capacity, GSH level and NADPH/NADP+ ratio in SNU886 cells (Fig. 2H) and SNU398 cells (Fig. S2E). These results suggest that mTOR improves the antioxidant ability of liver cancer cells and attenuates sorafenib-induced ferroptosis.

### mTOR-enhanced SESN3 promotes sorafenib resistance

**Fig. 3:**
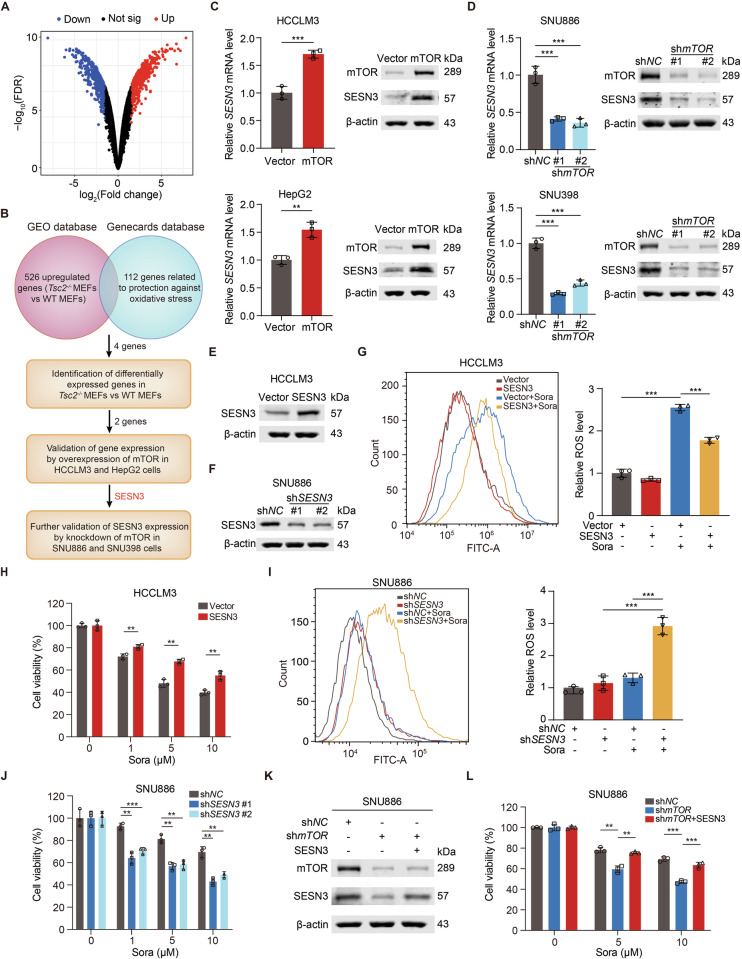
mTOR-enhanced SESN3 promotes sorafenib resistance. **A** Volcano plot exhibits the differentially expressed genes (DEGs) of *Tsc2*^*−/−*^ MEFs vs WT MEFs from GSE21755 dataset in GEO database. The thresholds were set as false discovery rate (FDR) < 0.01 and fold change >1.5. **B** Schematic delineation of flows and results of screening for potential candidates. **C**, **D** mRNA and protein levels of SESN3 in HCCLM3 or HepG2 cells (**C**) transfected with vector or mTOR plasmid, and SNU886 or SNU398 cells (**D**) transfected with control or mTOR shRNA. **E**, **F** Immunoblotting of HCCLM3 cells (**E**) transfected with vector or SESN3 plasmid, and SNU886 cells (**F**) transfected with control or SESN3 shRNA. **G**, **H** HCCLM3 cells were transfected with vector or SESN3 plasmid, *n* = 3.The intracellular ROS of cells treated with sorafenib (10 μM) for 24 h (**G**). Viability of cells treated with different concentrations of sorafenib for 24 h (**H**). **I**, **J** SNU886 cells were transfected with control or SESN3 shRNA, *n* = 3. The intracellular ROS of cells treated with sorafenib (10 μM) for 24 h (**I**). Viability of cells treated with different concentrations of sorafenib for 24 h (**J**). **K**, **L** SNU886/sh*mTOR* cells were transfected with vector or SESN3 plasmid. Immunoblotting of cells (**K**). Viability of cells treated with different concentrations of sorafenib for 24 h, *n* = 3 (**L**). Data are displayed as mean ± SD (error bars). **p < 0.01, ***p < 0.001. Sora: sorafenib.

To identify the potential key factor(s) responsible for sorafenib resistance in mTOR-activated cells, we analyzed differentially expressed genes (DEGs) between *Tsc2*^*−/−*^ MEFs and WT MEFs in GEO database (GSE21755) (Fig. 3A). The upregulated genes but not the downregulated genes were enriched in the pathways relevant to oxidoreductase activity, aldehyde dehydrogenase activity, and cellular responses to hypoxia (Fig. S3A, B). We then overlapped the upregulated genes in *Tsc2*^*−/−*^ MEFs with the genes associated with oxidative stress protection from the Genecards database and identified four potential candidates responsible for sorafenib resistance: *Pdk1, Gpx7, Sesn3*, and *Ak4* (Fig. 3B). qRT-PCR confirmed overexpression of *Sesn3* and *Pdk1* in *Tsc2*^*−/−*^ MEFs (Fig. S3C). Additionally, SESN3 but not PDK1 was increased at mRNA and protein levels in both HCCLM3/mTOR cells and HepG2/mTOR cells (Figs. 3C and S3D, E). In contrast, silencing mTOR decreased mRNA and protein of SESN3 in both SNU886 and SNU398 cells (Fig. 3D). Next, we sought to determine whether SESN3 was involved in the regulation of sorafenib resistance in mTOR-activated liver cancer cells. We overexpressed SESN3 in HCCLM3 cells and silenced SESN3 in SNU886 cells (Fig. 3E, F). Overexpressed SESN3 increased sorafenib resistance of HCCLM3 cells, as indicated by reduction of intracellular ROS and enhancement of cell viability (Fig. 3G, H). Conversely, silencing SESN3 enhanced sorafenib sensitivity by increasing intracellular ROS and decreasing the viability of SNU886 cells (Fig. 3I, J). To confirm sorafenib resistance of mTOR-activated liver cancer cells relying on high expression of SESN3, we overexpressed SESN3 in SNU886/sh*mTOR* cells (Fig. 3K). Overexpression of SESN3 reversed the impairment of sorafenib resistance caused by mTOR silencing, as evidenced by enhanced cell viability (Fig. 3L). SESN3 is thus positively regulated by mTOR and is critical for mTOR-mediated sorafenib resistance by reducing oxidative stress.

### CREB1 stimulates SESN3 transcription

**Fig. 4:**
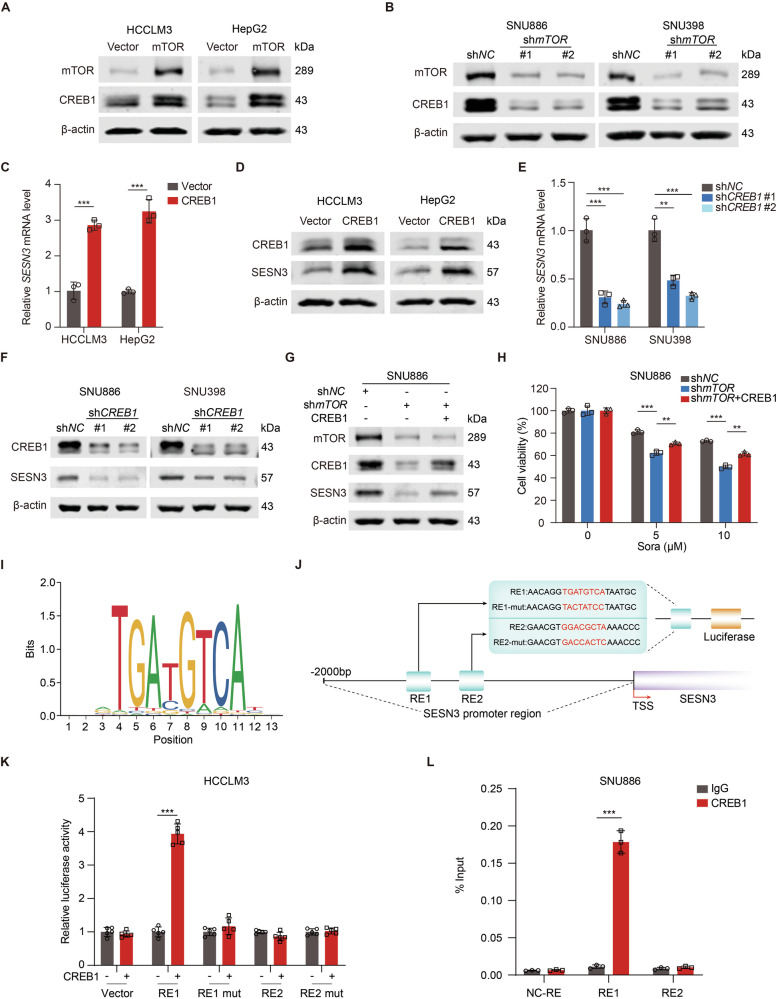
CREB1 stimulates SESN3 transcription. **A**, **B** Immunoblotting of HCCLM3 or HepG2 cells (**A**) transfected with vector or mTOR plasmid, and SNU886 or SNU398 cells (**B**) transfected with control or mTOR shRNA. **C**, **D** mRNA (**C**) and protein (**D**) levels of SESN3 in HCCLM3 and HepG2 cells transfected with vector or CREB1 plasmid. **E**, **F** mRNA (**E**) and protein (**F**) levels of SESN3 in SNU886 and SNU398 cells transfected with control or CREB1 shRNA. **G**, **H** SNU886/sh*mTOR* cells were transfected with vector or CREB1 plasmid. Immunoblotting of cells (**G**). Viability of cells treated with different concentrations of sorafenib for 24 h, *n* = 3 (**H**). **I** Sequence logo of CREB1 binding motif generated from JASPAR database. **J** Schematic representation of human SESN3 genomic structure. Shown are two potential CREB1 response elements (RE1 and RE2) and the corresponding mutant response elements (RE1 mut, RE2 mut). **K** Relative luciferase activity detected after transfection of luciferase reporter constructs containing RE1, RE2, RE1 mut, or RE2 mut into HCCLM3 cells. Renilla vector was used as a transfection internal control, *n* = 5. **L** Interaction between CREB1 and promoter region of SESN3 analyzed in SNU886 cells by CHIP assay. Three PCR probe sets were designed, namely RE1, RE2, and NC-RE, a negative control probe that is 5 kb upstream of the transcription start site of SESN3, *n* = 3. Data are displayed as mean ± SD (error bars). **p < 0.01, ***p < 0.001. Sora: sorafenib. TSS: transcription start site.

To determine whether mTOR activation of SESN3 expression is at the transcriptional level, we obtained SESN3 promoter sequence using UCSC genome browser and identified potential transcription factors in JASPAR database (Fig. S4A, B). Among them, CREB1 is a transcription factor that is positively regulated by mTOR in multiple biological processes [49]. CREB1 was enriched in both HCCLM3/mTOR and HepG2/mTOR cells (Fig. 4A). Conversely, silencing mTOR reduced CREB1 expression in SNU886 and SNU398 cells (Fig. 4B). Furthermore, CREB1 overexpression increased SESN3 mRNA and protein levels in HCCLM3 and HepG2 cells (Fig. 4C, D) while knockdown of CREB1 reduced SESN3 expression in SNU886 and SNU398 cells (Fig. 4E, F). Moreover, overexpressing CREB1 reversed the impairment of sorafenib resistance caused by mTOR silencing in SNU886 cells, as evidenced by enhanced cell viability (Fig. 4G, H). These findings suggest that CREB1 activation of SESN3 expression is critical for sorafenib resistance mediated by active mTOR.

To find out whether SESN3 is a transcriptional target of CREB1, JASPAR database was used to search for potential CREB1 response elements on the promoter region of SESN3 (Fig. 4I). Two putative CREB1 response elements (RE1, RE2) were identified with the highest predicted scores. Subsequently, we cloned sequences of wild-type response elements (RE1, RE2) and corresponding mutant response elements (RE1 mut, RE2 mut), respectively, into firefly luciferase reporter plasmids (Fig. 4J). Only RE1 but not RE2, RE1 mut, or RE2 mut induced luciferase expression in response to CREB1 overexpression in HCCLM3 cells (Fig. 4K). To check the potential direct interaction between CREB1 and SESN3 promoter, we performed a ChIP assay in SNU886 cells and observed that CREB1 was preferentially enriched in the RE1 genomic region (Fig. 4L). Therefore, CREB1 transcriptionally stimulates *SESN3* expression by directly binding to *SESN3* promoter.

### HSP70 facilitates the interaction between CREB1 and CREBBP

**Fig. 5:**
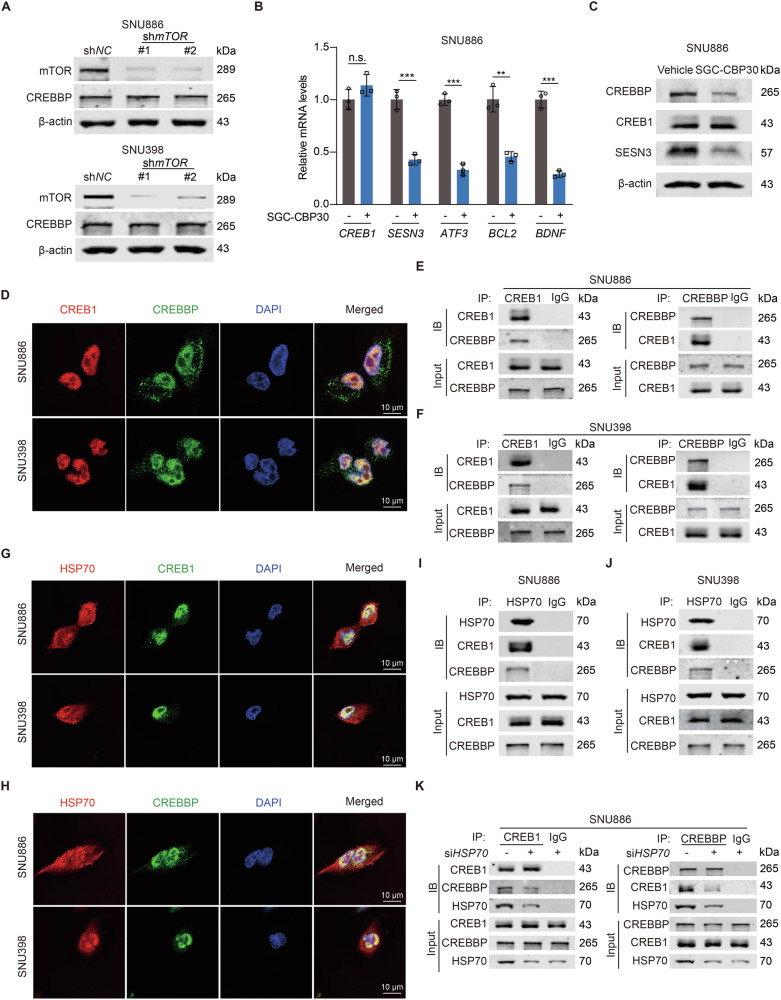
HSP70 facilitates the physical interaction between CREB1 and CREBBP. **A** Immunoblotting of SNU886 or SNU398 cells transfected with control or mTOR shRNA. **B**, **C** SNU886 cells were treated with SGC-CBP30 (5 μM) for 24 h. mRNA levels of CREB1 target genes, *n* = 3 (**B**). Immunoblotting of cells (**C**). **D** Confocal images illustrating the co-localization of CREB1 and CREBBP in SNU886 and SNU398 cells. Scale bar = 10 μm. **E**, **F** Co-IP demonstrating the interaction of CREB1 and CREBBP in SNU886 (**E**) and SNU398 (**F**) cells. **G**, **H** Confocal images showing the interaction of HSP70 with CREB1 (**G**) or CREBBP (**H**) in SNU886 and SNU398 cells. Scale bar = 10 μm. **I**, **J** Co-IP experiments showing the interaction of HSP70, CREB1, and CREBBP in SNU886 (**I**) and SNU398 (**J**) cells. **K** Co-IP analysis for CREB1 interaction with CREBBP after silencing HSP70 in SNU886 cells. Data are displayed as mean ± SD (error bars). n.s., not statistically significant, **p < 0.01, ***p < 0.001.

It has been documented that CREBBP, as a coactivator, participates in the transcriptional regulation of CREB1 [50]. To investigate the role of CREBBP in mTOR regulation of CREB1, we first assessed CREBBP expression in SNU886 and SNU398 cells with or without mTOR depletion. mTOR did not affect CREBBP expression (Fig. 5A). Furthermore, SGC-CBP30, a CREBBP inhibitor, reduced the mRNA levels of SESN3 and other CREB1 target genes, including *ATF3, BCL2 and BDNF* (Fig. 5B). CREBBP maintained CREB1 transcriptional activity without affecting CREB1 expression (Fig. 5C). To confirm the direct interaction between CREB1 and CREBBP, we performed immunofluorescent staining of SNU886 and SNU398 cells. CREB1 and CREBBP were colocalized in nuclei (Fig. 5D). The Co-IP experiment showed that CREBBP interacted with CREB1 in SNU886 and SNU398 cells (Fig. 5E, F). These results indicate that CREBBP probably binds to CREB1 and facilitates CREB1 transcriptional regulation in mTOR-activated cells.

Even though SGC-CBP30 hinders the transcriptional activity of CREB1 and has the potential to alleviate sorafenib resistance in mTOR-activated liver cancer, SGC-CBP30 is metabolized too quickly in vivo and might not be suitable for clinical application [51]. Therefore, we sought to identify other bioactive molecules with disruptive effects on CREB1 transcription. HSP70 is a ubiquitous chaperone and plays crucial roles in biological processes such as protein folding and assembly of protein complexes [52]. Supported by prediction from PPI network in STRING database, we speculated that HSP70 might participate in the assembly of CREB1/CREBBP complexes to influence CREB1-mediated transcription (Fig. S5). We thus investigated the subcellular location of HSP70 and its relationship with CREB1 and CREBBP in SNU886 and SNU398 cells. HSP70 was colocalized with both CREB1 and CREBBP in nuclei (Fig. 5G, H). Co-IP experiments further confirmed the interaction of HSP70 with CREB1/CREBBP complexes (Fig. 5I, J). Next, we silenced HSP70 in SNU886 cells to test whether HSP70 is necessary for the formation of CREB1/CREBBP complexes. Depleted HSP70 reduced co-precipitation of CREB1 and CREBBP in SNU886 cells (Fig. 5K). Transcriptional activity of CREB1 thus relies on the formation of CREB1/CREBBP complexes. HSP70 is crucial in maintaining the interaction between CREB1 and CREBBP.

### HSP70 inhibitor pifithrin-μ augments sorafenib-induced ferroptosis in mTOR-activated liver cancer cells

**Fig. 6:**
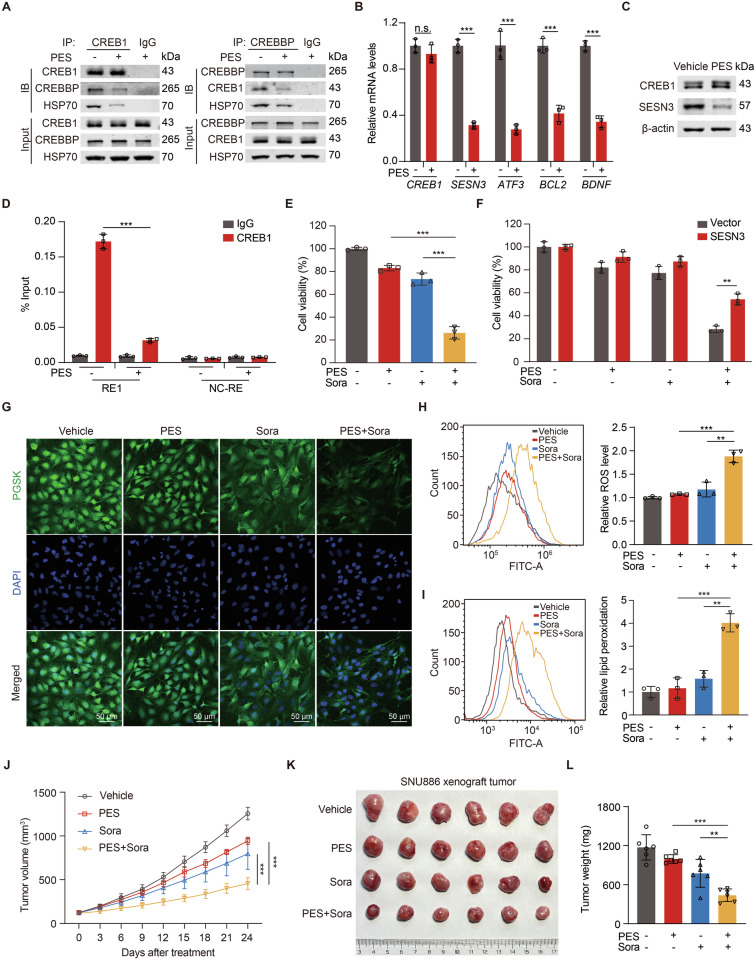
HSP70 inhibitor Pifithrin-μ augments sorafenib-induced ferroptosis in mTOR-activated liver cancer cells. **A**–**D** SNU886 cells were treated with pifithrin-μ (5 μM) for 24 h. Co-IP analysis demonstrates the interaction between CREB1, CREBBP, and HSP70 (**A**). mRNA levels of CREB1 target genes, *n* = 3 (**B**). Immunoblotting of cells (**C**). ChIP assay testing the recruitment of CREB1 to SESN3 genomic region RE1, *n* = 3 (**D**). **E**–**I** Concentration of pifithrin-μ and sorafenib were 5 μM and 10 μM, respectively. The treatment time was 24 h, *n* = 3. Viability of SNU886 (**E**) and SNU886 (**F**) cells transfected with vector or SESN3 plasmid. Representative confocal images using fluorescent PGSK dye, scale bar = 50 μm (**G**), relative ROS level (**H**), and lipid peroxidation (**I**) of SNU886 cells. **J**–**L** Nude mice were xenografted with SNU886 cells. Once tumor volume reached ~100 mm^3^, mice were treated with vehicle, pifithrin-μ (10 mg/kg, i.p.), sorafenib (20 mg/kg, i.g.), or combined pifithrin-μ (10 mg/kg, i.p.) and sorafenib (20 mg/kg, i.g.) every other day (*n* = 6 per group). Measurements of tumor volumes of nude mice every 3 days (**J**). Representative tumor images (**K**) and tumor weights (**L**) were recorded after sacrifice at the end of treatment. Data are displayed as mean ± SD (error bars). n.s., not statistically significant, **p < 0.01, ***p < 0.001. PES: pifithrin-μ, Sora: sorafenib.

To test whether HSP70 contributed to sorafenib resistance of mTOR-activated cells, we treated SNU886 cells with HSP70 inhibitor pifithrin-μ (PES). Pifithrin-μ disrupted the interaction between CREB1 and CREBBP (Fig. 6A) and reduced the mRNA levels of CREB1 target genes (Fig. 6B). Furthermore, pifithrin-μ suppressed SESN3 expression by disrupting the recruitment of CREB1 to genomic region of SESN3-RE1, but not NC-RE, a negative control probe which is 5 kb upstream of transcription start site of SESN3 (Fig. 6C, D). Moreover, pifithrin-μ potentiated the cell death caused by sorafenib in SNU886 cells (Fig. 6E). On the other hand, SESN3 overexpression canceled the lethality caused by combination of pifithrin-μ and sorafenib (Figs. 6F and S6A). To ascertain that pifithrin-μ and sorafenib-induced ferroptosis in mTOR-activated cells, we examined biomarkers of ferroptosis in SNU886 cells treated with pifithrin-μ and sorafenib. Compared to sorafenib alone, combined pifithrin-μ and sorafenib increased intracellular Fe^2+^ level illustrated as quenching of PGSK probe (Fig. 6G, Fig. S6B). Moreover, pifithrin-μ potentiated sorafenib-induced generation of intracellular ROS and lipid peroxidation in SNU886 cells (Fig. 6H, I). These in vitro findings prompted us to test the efficacy of this combinatory strategy in vivo. We injected SNU886 cells subcutaneously into nude mice. Combined pifithrin-μ and sorafenib treatment achieved greater inhibition of tumor growth than single drug application (Fig. 6J–L). There were no significant differences on body weights of nude mice with various combination of the two drugs (Fig. S6C). Taken together, pifithrin-μ suppresses CREB1/CREBBP complex formation, CREB1-mediated transcription, and sorafenib resistance of mTOR-activated liver cancer cells.

### Pifithrin-μ potentiates sorafenib sensitivity of mTOR-activated primary liver tumor

**Fig. 7:**
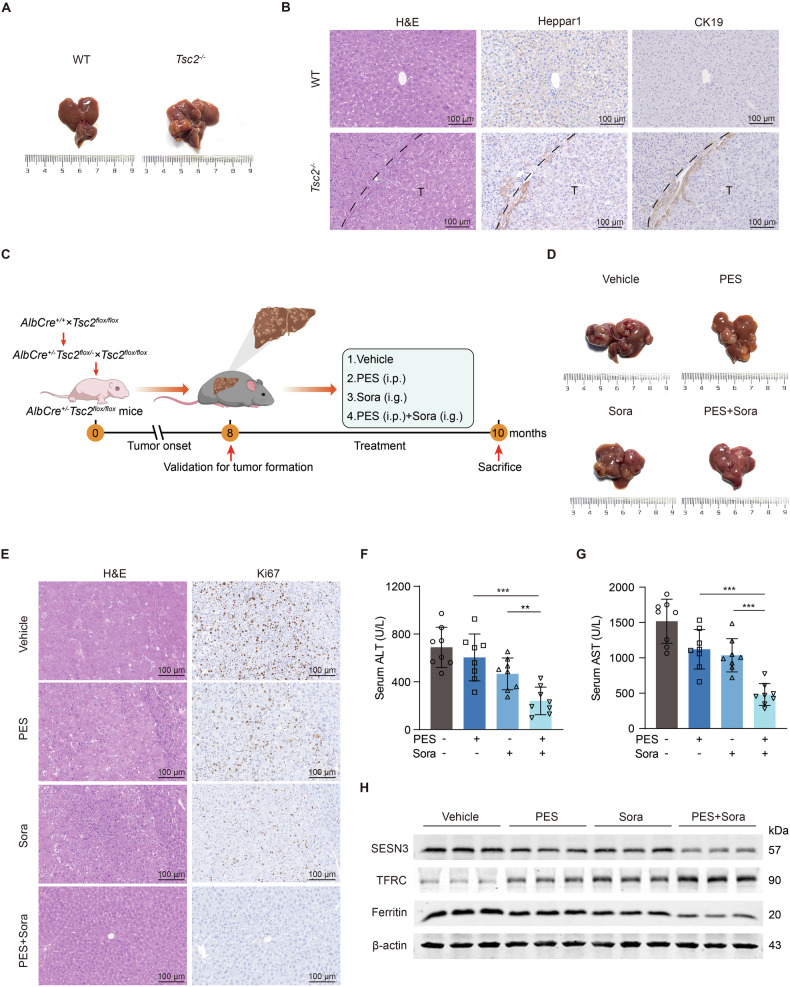
Pifithrin-μ potentiates sorafenib sensitivity of mTOR-activated primary liver tumor. **A**, **B** Tumor formation of 8-month-old mice. Representative liver images (**A**), liver tissue staining of H&E, Heppar1, and CK19, scale bar = 100 μm (**B**). **C**–**H** Liver tumors in *Tsc2*^*−/−*^ mice at 8 months. Mice were treated with vehicle, pifithrin-μ (10 mg/kg, i.p.), sorafenib (20 mg/kg, i.g.), or combined pifithrin-μ (10 mg/kg, i.p.) and sorafenib (20 mg/kg, i.g.) every other day (*n* = 8 per group) for 2 months. Schematic depiction of treatment schedules (**C**), representative liver images (**D**), liver tissue staining of H&E and Ki67, scale bar = 100 μm (**E**), serum ALT levels (**F**), serum AST levels (**G**), and immunoblotting (**H**). Data are displayed as mean ± SD (error bars). **p < 0.01, ***p < 0.001. PES pifithrin-μ, Sora sorafenib.

To test the effect of pifithrin-μ and sorafenib on mTOR-activated liver cancer development, we bred *Tsc2*-*floxed* mice with *Alb-Cre* transgenic mice to generate *Tsc2*^flox/flox^; *Alb*^*cre*^ mice (a.k.a. *Tsc2*^−/−^) (Fig. S7A, B). At 8 months of age, macroscopic liver tumors were observed in *Tsc2*^*−/−*^ mice but not in WT mice (Fig. 7A). *Tsc2*^−/−^ mice had disordered liver tissue structures, manifested by the lack of clear hepatic lobules. Positive Heppar1 and negative CK19 stainings indicated that the tumors were HCC (Fig. 7B). We treated *Tsc2*^−/−^ mice with pifithrin-μ and sorafenib for two months (Fig. 7C). This combination regimen exerted synergistic suppression on tumor numbers, liver-to-body weight ratios, serum ALT and AST levels as compared to sorafenib or pifithrin-μ alone (Figs. 7D–G and S7D–E), without significant impact on body weights of mice (Fig. S7C). In addition, combined treatment reduced SESN3 expression in mouse liver tumors, accompanied by Fe^2+^ accumulation (Fig. 7H). These data suggest that combined treatment of pifithrin-μ and sorafenib inhibits liver tumor advancement of *Tsc2*^−/−^ mice.

## Discussion

**Fig. 8:**
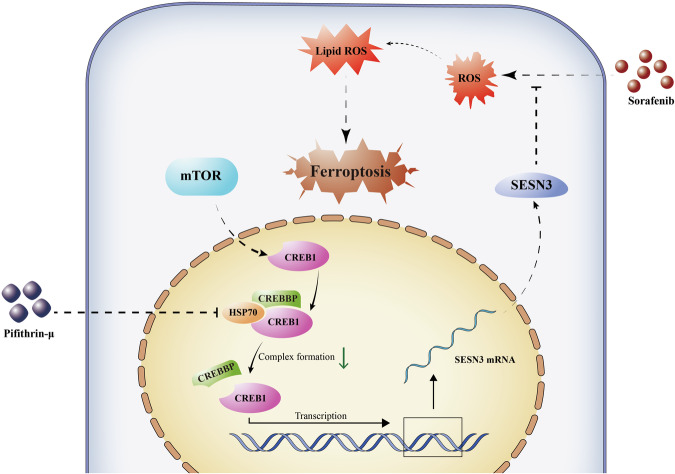
Illustration of pifithrin-μ enhancing sorafenib efficacy by targeting mTOR/CREB1/SESN3 axis in liver cancer cells.

As a multi-target tyrosine kinase inhibitor, sorafenib has been used in the clinic for stabilizing HCC progression by inducing oxidative stress. However, different etiological factors of HCC may contribute to various responses of cells to sorafenib [10]. In this study, we observed that mTOR-activated cells were resistant to sorafenib treatment. Mechanistically, HSP70 potentiated CREB1 transcription to participate in SESN3-mediated antioxidant capacity in mTOR-activated cells. HSP70 inhibitor pifithrin-μ enhanced the efficacy of sorafenib to repress mTOR-activated tumorigenesis (Fig. 8).

mTOR signaling pathway is activated in a subtype of HCC [28, 53]. Notably, TSC1 and TSC2 emerge as the most frequently mutated genes linked to activated mTOR signaling in HCC tumor samples. We found that mTOR-activated liver cancer cells were resistant to sorafenib-induced ferroptosis. Targeting the mTOR signaling pathway is thus expected to be an important strategy to reverse sorafenib resistance for liver cancer. However, a combination of sorafenib and everolimus did not prolong the survival of HCC patients [40, 41]. Therefore, exploring the mechanism of mTOR resistance to sorafenib is crucial for improving targeted therapy for liver cancer.

Heightened ROS levels are believed to hinder tumor growth. ROS interacts with polyunsaturated fatty acids in lipid membranes, resulting in lipid ROS formation and triggering ferroptosis. Targeting ROS thus represents a promising therapeutic strategy. However, tumors possess an inherent antioxidant capacity that enables them to combat oxidative stress and augment their resistance to drugs. In HCC, specific antioxidant enzymatic genes are overexpressed, contributing to sorafenib resistance [54]. We observed that mTOR activation results in sorafenib resistance in liver cancer cells by enhancing cellular antioxidant capacity. These findings are corroborated by studies indicating a mechanistic connection between mTOR and redox balance [55].

It is well established that SESN3 encodes antioxidant modulators of peroxiredoxins, participating in the maintenance of redox homeostasis [56]. We demonstrated that SESN3 was a target gene of mTOR and played a crucial role in conferring active mTOR cells resistance to sorafenib. CREB1, a transcription factor governing genes linked to both cell survival and apoptosis, undergoes regulation by numerous protein kinases and phosphatases [57]. Previously, we identified that activation of mTOR induced CREB1 phosphorylation and its subsequent accumulation [49]. Here, CREB1 enhanced SESN3 expression by binding to SESN3 promoter and subsequently boosted the antioxidant capacity of liver cancer cells.

CREB1 operates as a cAMP-regulated transcription factor that stimulates target gene expression, partly through interaction with coactivator paralogs such as CREBBP. We observed that HSP70 facilitated the binding of CREB1 and CREBBP. The chaperone role of HSP70 involves binding to folded proteins and inducing conformational changes that impact PPIs [58]. Additionally, HSP70 has the potential to serve as a sensitive marker for distinguishing early HCC from precancerous lesions or noncancerous liver conditions [59]. Pifithrin-μ functions by interacting with the substrate-binding domain of the HSP70 carboxyl-terminal and disrupting the association between HSP70 and its co-chaperones. This inhibitor has exhibited cytotoxic effects on various types of tumor cells including acute leukemia, bladder cancer cells, and prostate cancer cells, with little toxicity towards normal cells [60]. However, its efficacy in the treatment of liver cancer has not been tested yet. In our study, pifithrin-μ inhibited HSP70 to reduce the transcriptional activity of CREB1 and the expression of SESN3, consequently suppressing liver cancer cell proliferation and tumorigenesis.

Current combination therapies, such as radiotherapy, cytotoxic chemotherapy, and molecular targeted therapy, may overcome sorafenib resistance and improve the effectiveness of sorafenib [8, 12]. Our study reveals that pifithrin-μ synergizes with sorafenib to suppress the proliferation and tumorigenesis of mTOR-activated cells. This innovative combination therapy presents a compelling strategy to enhance therapeutic efficacy of sorafenib.

In conclusion, activated mTOR confers cells resistance to sorafenib treatment by increasing cellular antioxidant capacity through CREB1/CREBBP/HSP70-induced SESN3 expression. Pifithrin-μ in conjunction with sorafenib exerts the therapeutic potential in the treatment of TSC2-deficient human liver cancer cell-derived xenograft tumors and spontaneous mouse liver cancer. Targeting mTOR-CREB1-SESN3 axis may offer a promising therapeutic strategy to alleviate sorafenib resistance of TSC2 deficiency-associated mTOR-activated liver cancer.

## Supplementary information

Supplementary materials

Full and uncropped western blots

## Data Availability

The data analyzed in this study are included in both the published article and the supplemental data files.
