# Supplementary material for: Pifithrin-μ sensitizes mTOR-activated liver cancer to sorafenib treatment
Source: Cell Death Dis. 2025 Jan 26;16(1):42. doi: 10.1038/s41419-025-07332-6 (PMC11762308; doi:10.1038/s41419-025-07332-6)
Supplement: Supplementary file 1 — Supplementary materials [file 41419_2025_7332_MOESM1_ESM.docx]

**Pifithrin-μ sensitizes mTOR-activated liver cancer to sorafenib treatment**

**Running title:** HSP70 inhibitor attenuates mTOR resistance to sorafenib

Jiarui Lv^1,2^, Yanan Wang^2^, Jiacheng Lv^3^, Cuiting Zheng^2^, Xinyu Zhang^4^, Linyan Wan^5^, Jiayang Zhang^6^, Fangming Liu^2^, Hongbing Zhang^1,2^

^1^Department of Organ Transplantation and Hepatobiliary Surgery, Key Laboratory of Organ Transplantation of Liaoning Province, The First Hospital of China Medical University, Shenyang, China.

^2^Department of Physiology, State Key Laboratory of Common Mechanism Research for Major Diseases, Haihe Laboratory of Cell Ecosystem, Institute of Basic Medical Sciences and School of Basic Medicine, Chinese Academy of Medical Sciences and Peking Union Medical College, Beijing, China.

^3^Department of Plastic Surgery, The First Hospital of China Medical University, Shenyang, China.

^4^Department of Radiology, State Key Laboratory of Complex, Severe and Rare Diseases, Chinese Academy of Medical Sciences, Peking Union Medical College and Peking Union Medical College Hospital, Beijing, China.

^5^Department of Gastroenterology, Yichang Central People’s Hospital, The First College of Clinical Medical Science, China Three Gorges University, Yichang, China.

^6^Department of Breast Oncology, Key Laboratory of Carcinogenesis and Translational Research, Peking University Cancer Hospital and Institute, Beijing, China.

**Corresponding author:** Hongbing Zhang, MD, PhD, [hbzhang@ibms.pumc.edu.cn](mailto:hbzhang@ibms.pumc.edu.cn)

**Supplementary materials**

**
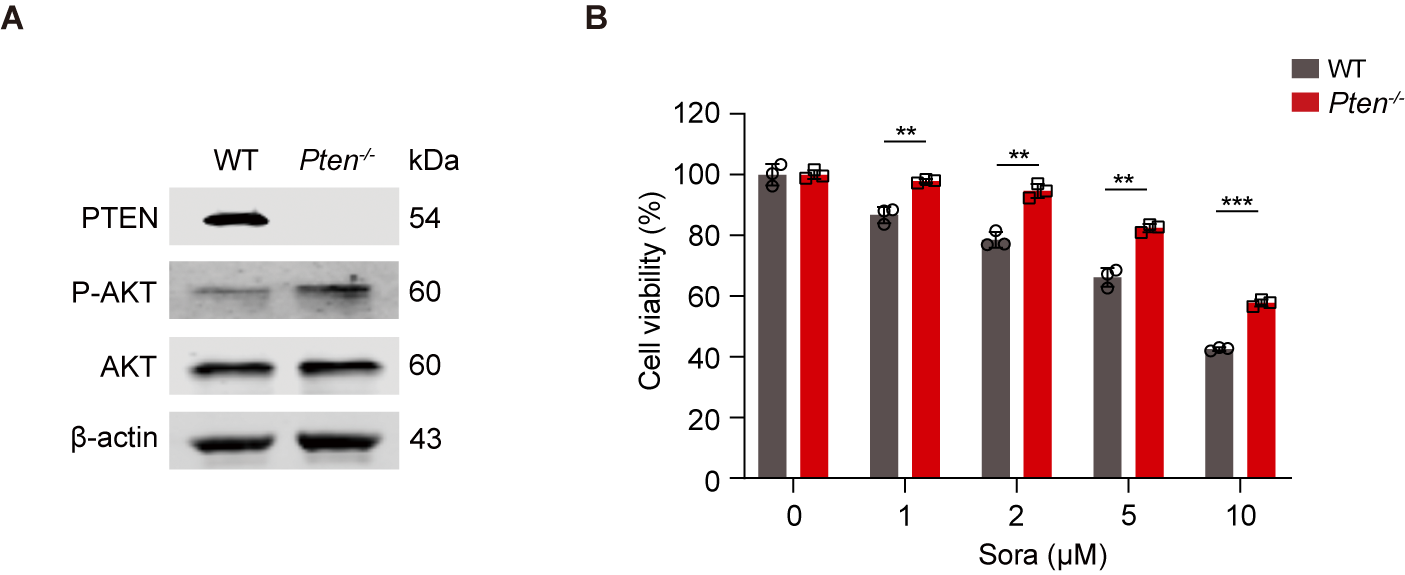
**

**Fig. S1. mTOR activation confers cell resistance to sorafenib.**

**(A)** Immunoblotting of *Pten*^-/-^ MEFs. **(B)** Viability of *Pten*^-/-^ MEFs treated with different concentrations of sorafenib for 24 h, n=3. Data are displayed as mean ± SD (error bars). **p<0.01, ***p<0.001. Sora: sorafenib.

**Fig. S2.** **mTOR attenuates sorafenib-mediated ROS accumulation and oxidative stress in liver cancer cells.**


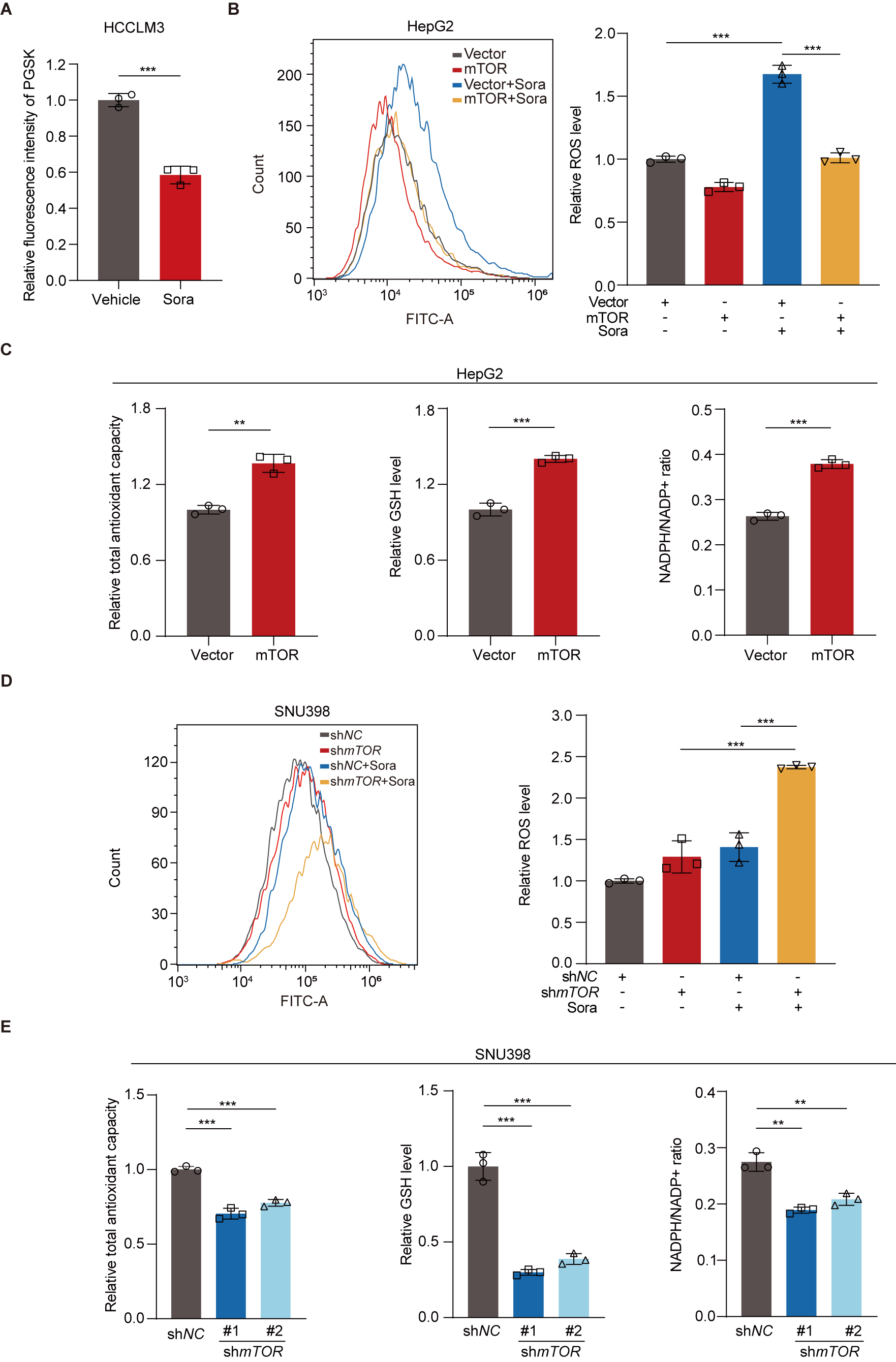


(**A**) Relative PGSK fluorescence intensity was assessed in HCCLM3 cells after treatment with sorafenib (10 μM) for 24 h, n=3. (**B, C**) HepG2 cells were transfected with vector or mTOR plasmid and treated with sorafenib (10 μM) for 24 h, n=3. The intracellular ROS were measured with flow cytometry (**B**). Relative total antioxidant capacity, GSH level, and NADPH/NADP+ ratio were measured (**C**). (**D, E**) SNU398 cells were transfected with control or mTOR shRNA and treated with sorafenib (10 μM) for 24 h, n=3. The intracellular ROS were measured with flow cytometry (**D**). Relative total antioxidant capacity, GSH level, and NADPH/NADP+ ratio were measured (**E**). Data are displayed as mean ± SD (error bars). **p<0.01, ***p<0.001. Sora: sorafenib.


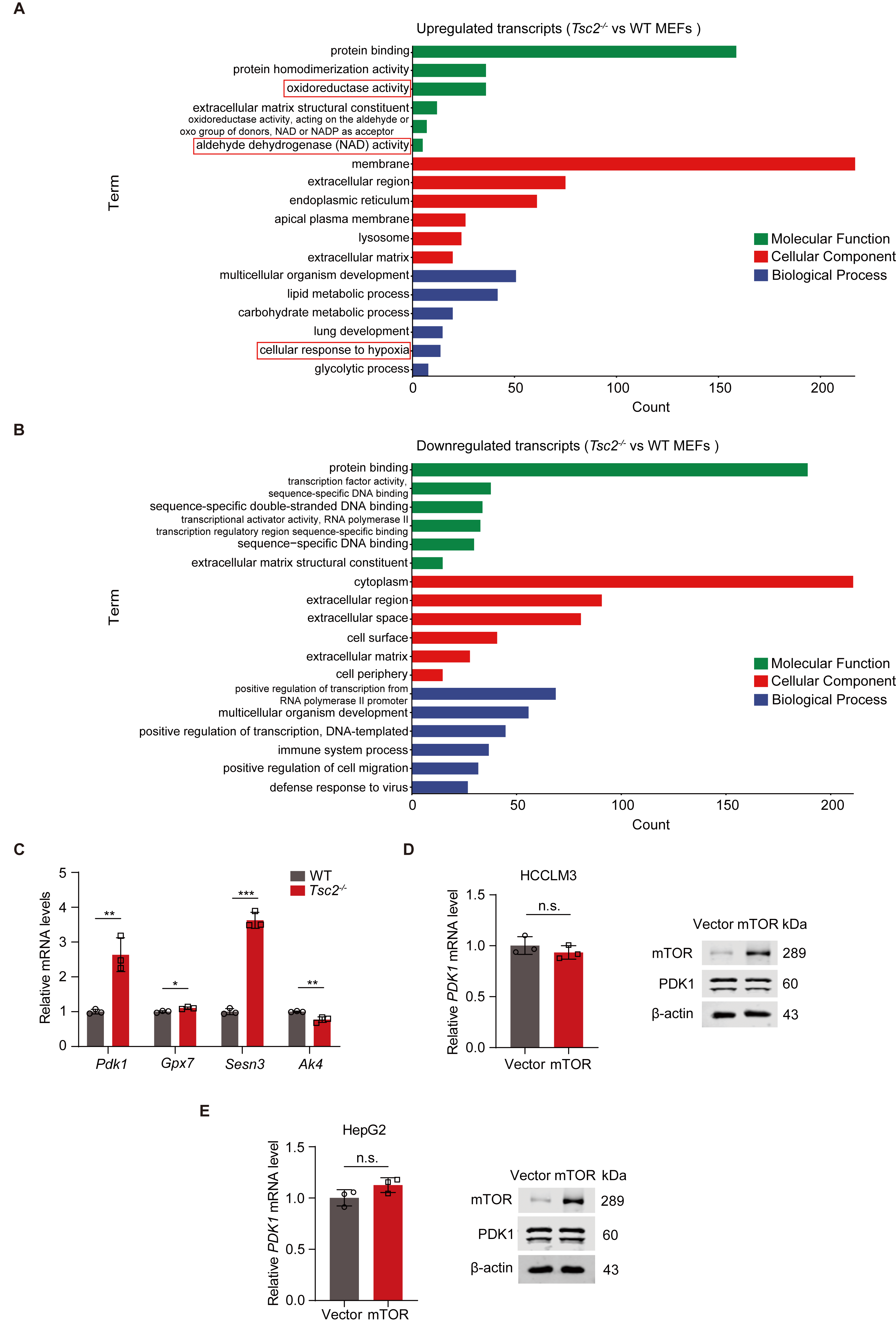
**Fig. S3.** **mTOR-enhanced SESN3 promotes sorafenib resistance.**

(**A, B**) GO enrichment analysis of the differentially expressed genes (DEGs) between *Tsc2*^-/-^ MEFs and WT MEFs was performed for both upregulated (**A**) and downregulated (**B**) transcripts. (**C**) Validation of differentially expressed mRNAs in *Tsc2^-/-^* MEFs, n=3. (**D, E**) mRNA and protein levels of PDK1 in HCCLM3 (**D**) or HepG2 (**E**) cells transfected with vector or mTOR plasmid. Data are displayed as mean ± SD (error bars). n.s., not statistically significant, **p<0.01, ***p<0.001.

**
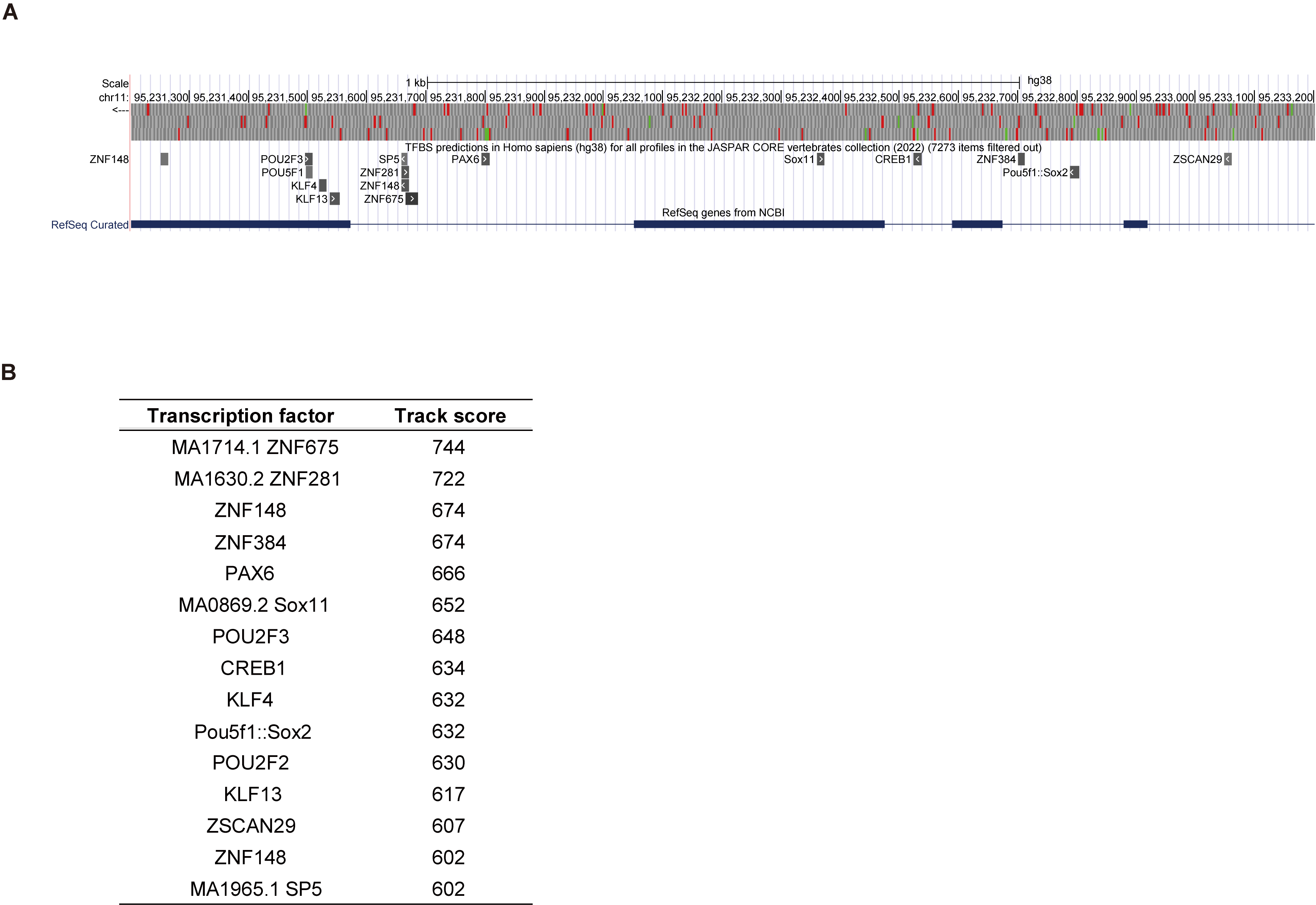
**

**Fig. S4.** Predicted potential transcription factors (**A**) and corresponding scores (**B**) in the SESN3 promoter region using JASPAR transcription factor binding sites database.


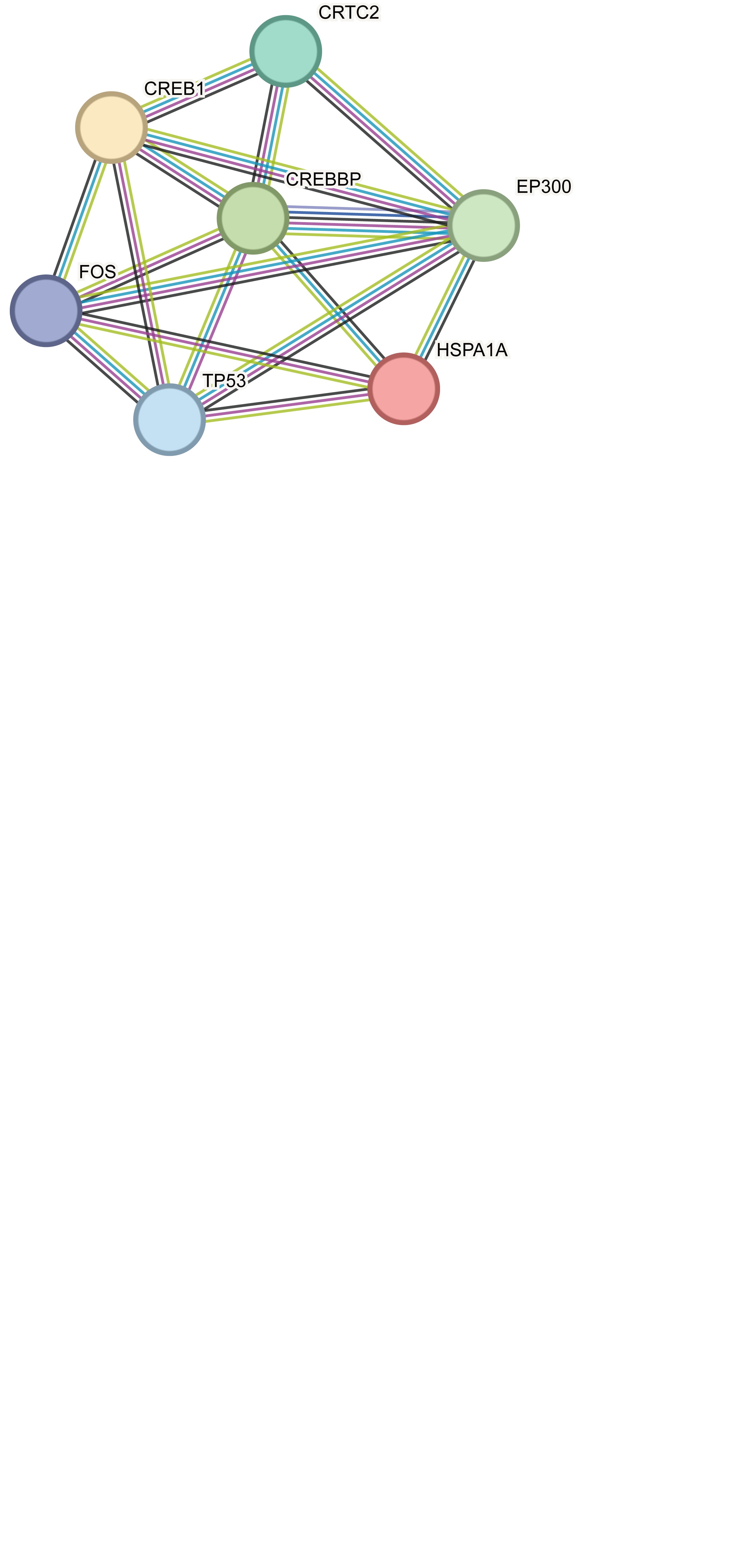


**Fig. S5.** PPI analysis reveals the interaction between CREB1, CREBBP and HSPA1A (HSP70) using the STRING website.


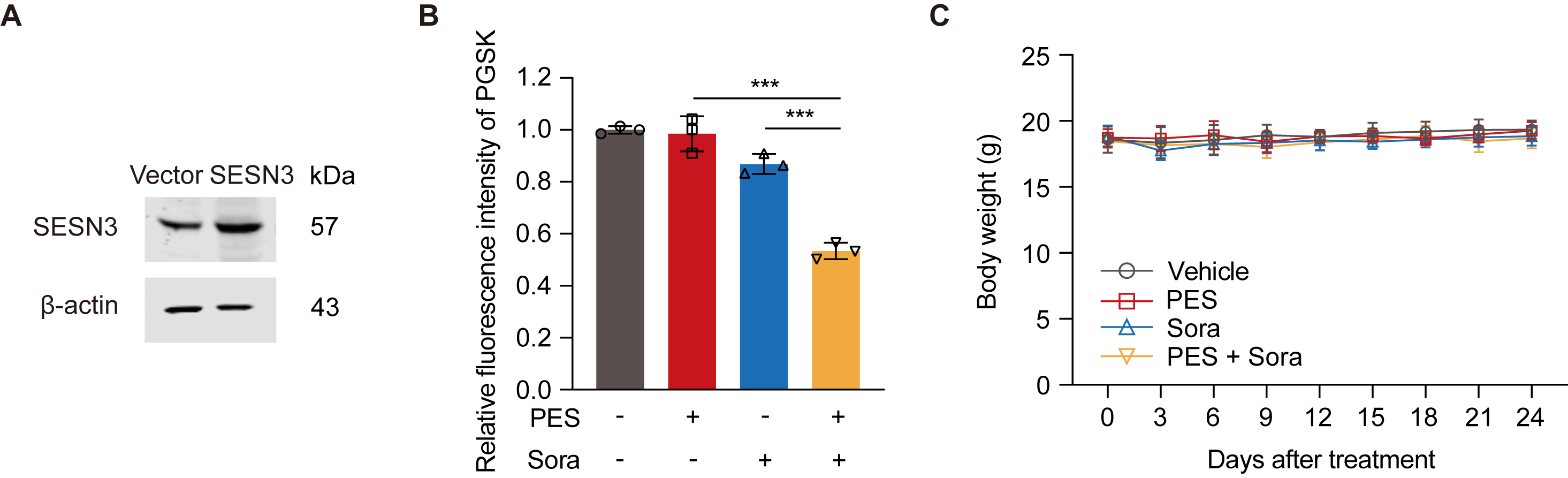
**Fig. S6. HSP70 inhibitor pifithrin-μ augments sorafenib-induced ferroptosis in mTOR-activated liver cancer cells.**

(**A**) Immunoblotting of SNU886 cells transfected with vector or SESN3 plasmid. (**B**) Relative PGSK fluorescence intensity changes were assessed in SNU886 cells after treatment with pifithrin-μ (5 μM) and sorafenib (10 μM) for 24 h, n=3. (**C**) Nude mice were inoculated with SNU886 cells. Once tumor volume reached around 100 mm^3^, mice were treated with vehicle, pifithrin-μ (10 mg/kg, i.p.), sorafenib (20 mg/kg, i.g.), or combined pifithrin-μ (10 mg/kg, i.p.) and sorafenib (20 mg/kg, i.g.) every other day (n=6 per group). Measurements of body weight every 3 days. Data are displayed as mean ± SD (error bars). ***p<0.001. Sora: sorafenib. PES: pifithrin-μ.


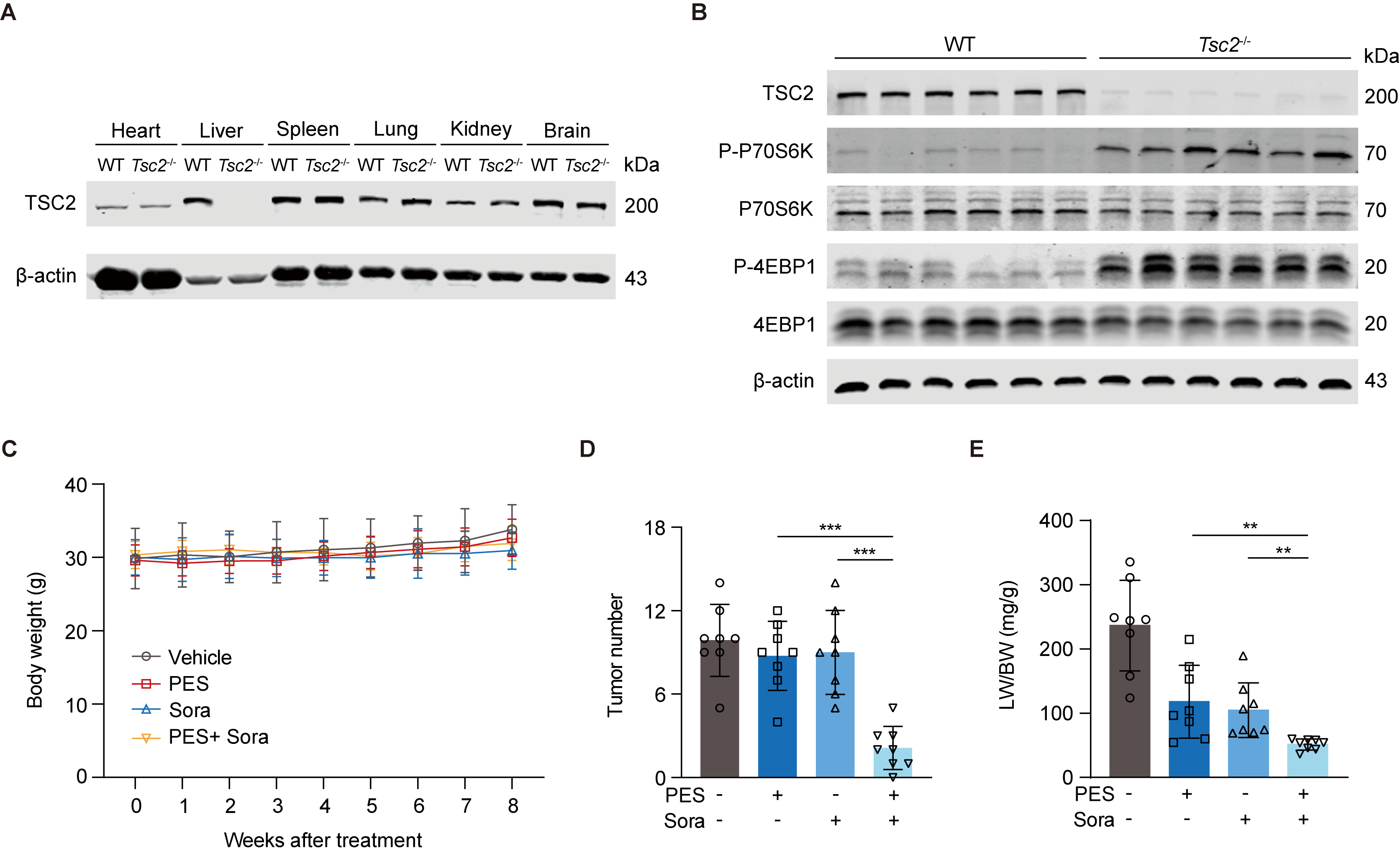


**Fig. S7. Pifithrin-μ potentiates sorafenib sensitivity of mTOR-activated primary liver tumor.**

(**A, B**) Immunoblotting of organs (**A**) and liver (**B**) of WT and *Tsc2^-/-^* mice. (**C-E**) 8-month-old mice were treated with vehicle, pifithrin-μ (10 mg/kg, i.p.), sorafenib (20 mg/kg, i.g.), or combined pifithrin-μ (10 mg/kg, i.p.) and sorafenib (20 mg/kg, i.g.) every other day (n=8 per group) for 2 months. Body weight (**C**), tumor numbers (**D**), and liver weight-to-body weight ratios (**E**). Sora: sorafenib. PES: pifithrin-μ.
