# Supplementary material for: Pifithrin-μ sensitizes mTOR-activated liver cancer to sorafenib treatment
Source: Cell Death Dis. 2025 Jan 26;16(1):42. doi: 10.1038/s41419-025-07332-6 (PMC11762308; doi:10.1038/s41419-025-07332-6)
Supplement: Supplementary file 2 — Full and uncropped western blots [file 41419_2025_7332_MOESM2_ESM.docx]

**Figure 1**

**A**


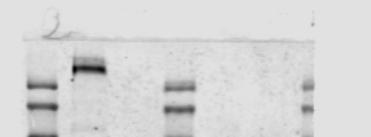

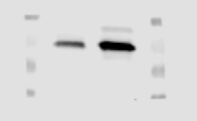

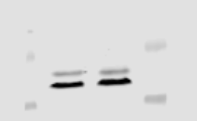

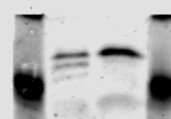

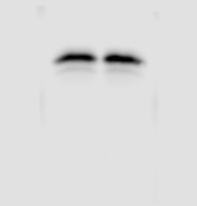

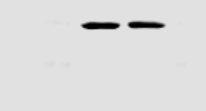


**TSC2 P-P70S6k P70S6k P-4EBP1 4EBP1 β-actin**

**C**


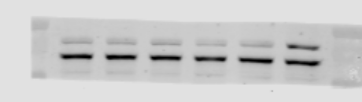

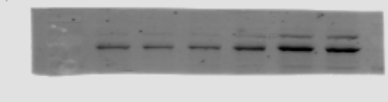

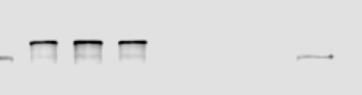


**TSC2 P-P70S6k P70S6k**


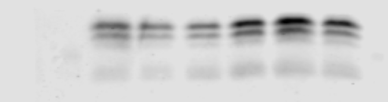

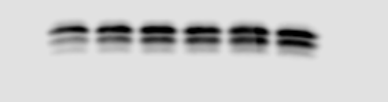

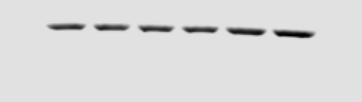


**P-4EBP1 4EBP1 β-actin**

**E**


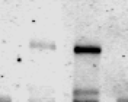

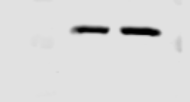


**mTOR β-actin**

**F**


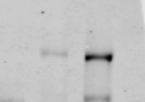

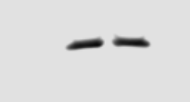


**mTOR β-actin**

**G**


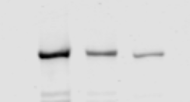

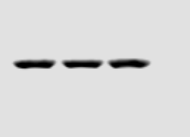


**mTOR β-actin**

**H**


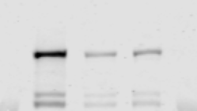

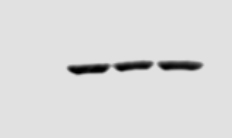


**mTOR β-actin**

**Figure 3**

**C**


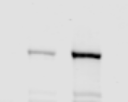

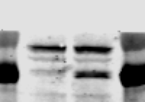

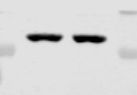

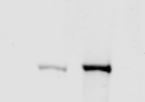

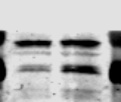

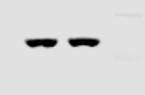


**mTOR SESN3 β-actin mTOR SESN3 β-actin**

**D**


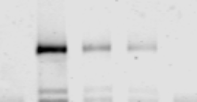

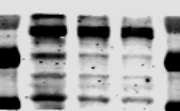

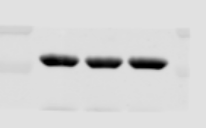


**mTOR SESN3 β-actin**


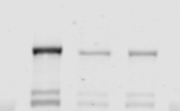

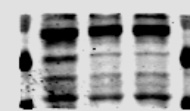

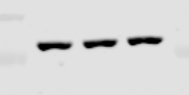


**mTOR SESN3 β-actin**

**E**


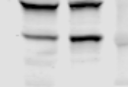

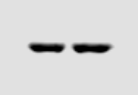


**SESN3 β-actin**

**F**


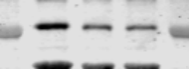

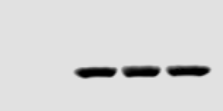


**SESN3 β-actin**

**K**


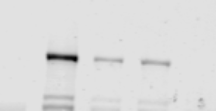

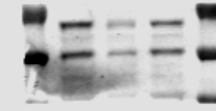

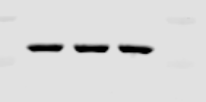


**mTOR SESN3 β-actin**

**Figure 4**

**A**


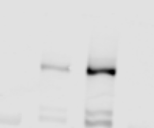

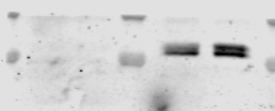

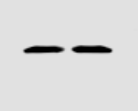


**mTOR CREB1 β-actin**


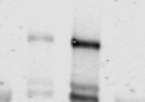

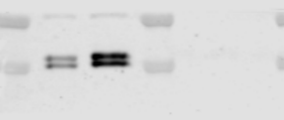

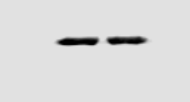


**mTOR CREB1 β-actin**

**B**


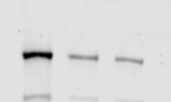

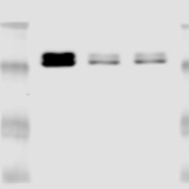

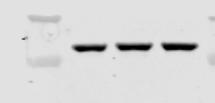


**mTOR CREB1 β-actin**


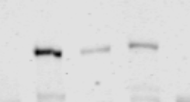

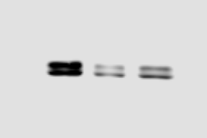

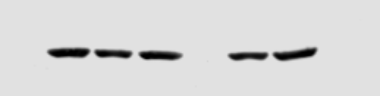


**mTOR CREB1 β-actin**

**D**


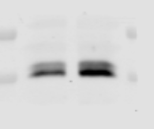

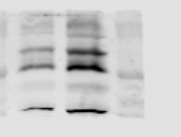

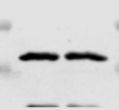


**CREB1 SESN3 β-actin**


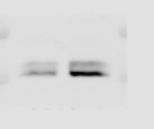

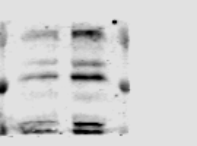

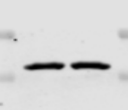


**CREB1 SESN3 β-actin**

**F**


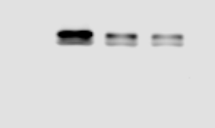

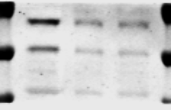

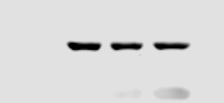


**CREB1 SESN3 β-actin**


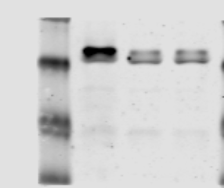

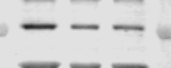

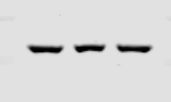


**CREB1 SESN3 β-actin**

**G**


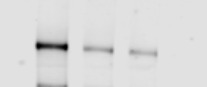

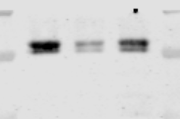

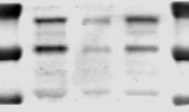

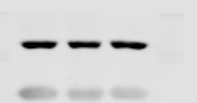


**mTOR CREB1 SESN3 β-actin**

**Figure 5**

**A**


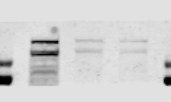

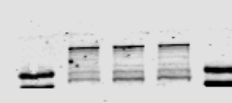

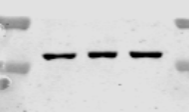


**mTOR CREBBP β-actin**


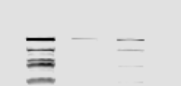

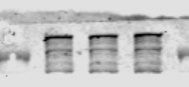

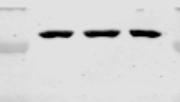


**mTOR CREBBP β-actin**

**C**


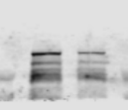

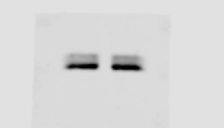

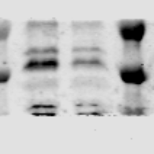

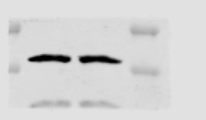


**CREBBP CREB1 SESN3 β-actin**

**E**


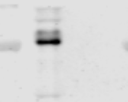

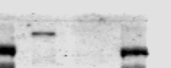

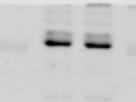

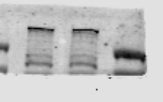


**CREB1 CREBBP CREB1 CREBBP**


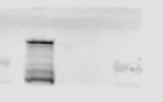

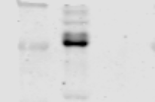

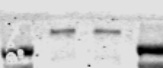

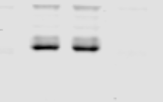


**CREBBP CREB1 CREBBP CREB1**

**F**


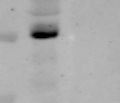

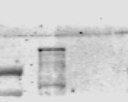

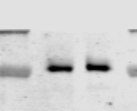

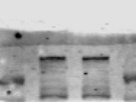


**CREB1 CREBBP CREB1 CREBBP**


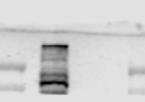

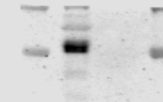

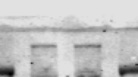

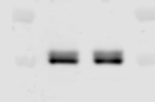


**CREBBP CREB1 CREBBP CREB1**

**I**


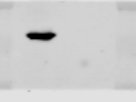

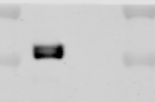

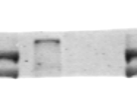

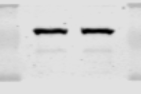

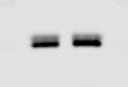

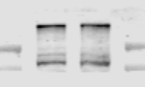


**HSP70 CREB1 CREBBP HSP70 CREB1 CREBBP**

**J**


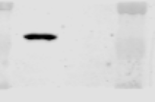


**HSP70 CREB1 CREBBP HSP70 CREB1 CREBBP**

**K**

**CREB1 CREBBP HSP70 CREB1 CREBBP HSP70**

**CREBBP CREB1 HSP70 CREBBP CREB1 HSP70**

**Figure 6**

**A**

**CREB1 CREBBP HSP70 CREB1 CREBBP HSP70**

**CREBBP CREB1 HSP70 CREBBP CREB1**

**HSP70**

**C**

**CREB1 SESN3 β-actin**

**Figure 7**

**SESN3**

**TFRC**

**Ferritin**

**β-actin**

**Fig. S1**

**PTEN P-AKT AKT β-actin**

**Fig. S3**

**D**

**mTOR PDK1 β-actin**

**E**

**mTOR PDK1 β-actin**

**Fig. S6**

**A**

**SESN3 β-actin**

**Fig. S7**

**A**

**TSC2**

**β-actin**

**B**

**TSC2**

**P-P70S6k**

**P70S6k**

**P-4EBP1**

**4EBP1**

**β-actin**
